# Antibacterial and Antifungal Activities of Ethiopian Medicinal Plants: A Systematic Review

**DOI:** 10.3389/fphar.2021.633921

**Published:** 2021-06-01

**Authors:** Dereje Nigussie, Gail Davey, Takele Beyene Tufa, Malcolm Brewster, Belete Adefris Legesse, Abebaw Fekadu, Eyasu Makonnen

**Affiliations:** ^1^ Centre for Innovative Drug Development and Therapeutic Trials for Africa (CDT-Africa), College of Health Sciences, Addis Ababa University, Addis Ababa, Ethiopia; ^2^ Centre for Global Health Research, Brighton and Sussex Medical School, University of Sussex, Brighton, United Kingdom; ^3^ School of Public Health, Addis Ababa University, Addis Ababa, Ethiopia; ^4^ Department of Biomedical Sciences, College of Veterinary Medicine and Agriculture, Addis Ababa University, Addis Ababa, Ethiopia; ^5^ Rye Medical Centre, Rye, United Kingdom; ^6^ Department of Pharmacology and Clinical Pharmacy, College of Health Sciences, Addis Ababa University, Addis Ababa, Ethiopia

**Keywords:** systematic literature review, antifungal, antibacterial, Ethiopia, medicinal plants

## Abstract

**Background:** Podoconiosis and lymphatic filariasis are the most common causes of lower limb lymphoedema in the tropics. Many sufferers experience frequent painful episodes of acute bacterial infection. Plant based traditional medicines are used to treat infections in many countries and are culturally established in Ethiopia. Ethiopian medicinal plants found to have antibacterial and antifungal activities were reviewed with the aim of increasing information about the treatment of wound infections in patients with lymphoedema.

**Methods:** This study collates data from published articles on medicinal plants with antibacterial and antifungal activities in Ethiopia. A systematic search of Scopus, EMBASE, PUBMED/MEDLINE and Google Scholar was undertaken. The Preferred Reporting Items for Systematic Reviews and Meta-analysis (PRISMA) guidelines were followed. The protocol was registered on PROSPERO with registration number CRD42019127471. All controlled studies of *in vitro* antibacterial and antifungal activities were considered. All articles containing the descriptors published until June 28, 2019 were included. The outcome was measured as percent inhibition of microbial growth. For quality assessment of individual *in vitro* studies, OECD guidelines and the WHO-Good Laboratory Practice (GLP) handbook were used.

**Results:** Seventy-nine studies met the inclusion criteria. A total of 150 plant species and three compounds had been tested against 42 species of bacteria, while 43 plant species had been tested against 22 species of fungus.

**Conclusion:** Materials derived from several Ethiopian medicinal plants have been shown to have promising activity against a variety of bacteria and fungi. Those derived from *Azadiractha indica* A. Juss. and *Lawsonia inerms* L*.* are the most extensively studied against a wide range of gram-negative and positive bacteria, and fungal species.

## Introduction

Lymphoedema is a chronic illness that has a major physical and psychological effect on patients and lowers the quality of patient life substantially. Neglected tropical diseases (NTDs) such as podoconiosis, lymphatic filariasis, and leprosy are the most common causes of lower limb lymphoedema in the tropics. Suffering can be aggravated by frequent painful episodes of acute bacterial limb infection known as “acute attacks” (Ndzeshang et al., 2020). Cellulitis and erysipelas are typical wound complications, with the majority of infections caused by group A, C, or G streptococci and *Staphylococcus aureus* species (Al-Niaimi and Cox, 2009). However, *Aeromonas hydrophila/caviae, Acinetobacter lwoffii, Escherichia coli, Klebsiella pneumoniae, Pseudomonas aeruginosa, Shewanella algae, Staphylococcus aureus, Streptococcus pyogenes, Streptococcus dysgalactiae, Staphylococcus haemolyticus, Streptococcus agalactiae* and *Staphylococcus simulans* have recently been found to be involved in colonising wounds of lymphoedematous limbs in patients from Ethiopia (Nigussie et al., 2020b).

Lymphoedema caused by the aforementioned NTDs is progressive if not treated. In the early stages oedema can be reversed overnight, but with disease progression there can be serious impairment and loss of independence. Patients are excluded from society because of their incapacitating and stigmatized impairments which cause significant economic effects, intergenerational poverty, and alienation from society. In Ethiopia, an estimate of 5.6 and 34.9 million peoples are at risk of lymphatic filariasis and podoconiosis respectively (Caprioli et al., 2020). There are 1.5 million cases of podoconiosis across 345 districts of Ethiopia, and the country has the highest prevalence of podoconiosis in the world (Deribe et al., 2020).

The use of medicinal plants has a long history in the treatment of a range of diseases, including infectious diseases, and these days hundreds of thousands of plant species have been tested for their medicinal properties (Górniak et al., 2019). However, the phytochemical and pharmacological activities of many more plants remain to be studied. Plant-derived substances are tolerated and accepted by patients and seem a reliable source of antimicrobial compounds (Górniak et al., 2019).

Medicinal plants are commonly used worldwide as alternative treatments for mental and physical illnesses. Herbal formulated medicines and traditional health practices are considered more affordable and accessible to most rural societies than modern drugs (Jima and Megersa, 2018). The World Health Organization (WHO) estimated that about 65% of the world population use medicinal plants for their primary health care. In addition, approximately 39% of the drugs developed since 1980 have been derived from plants and their derivatives (Nwonuma et al., 2020).

Traditional medicine has long been established in the culture of Ethiopian communities (Lulekal et al., 2014). In rural areas this includes the use of plant-based treatments of inflammation, wounds and infection (Gebremeskel et al., 2018). Records from as far back as the fifteenth century detail traditional medical practices and remedies obtained from oral tales, early medico-religious manuscripts, and traditional pharmacopeia (Jima and Megersa, 2018). The antimicrobial activities of these traditional medicinal plants are based on their secondary metabolites such as alkaloids, terpenoids, flavonoids, tannins and glycosides (Sisay et al., 2019).

Many *in vitro* antibacterial and antifungal studies have been conducted on the safety and efficacy of Ethiopian medicinal plants used to treat bacterial and fungal infections. However, data on the efficacy and safety of these medicinal plants in the management of wound infection have not yet been summarized. This systematic review draws together up-to-date information on Ethiopian medicinal plants used as antibacterial and antifungal agents that might potentially be used for the management of wound infections in lymphoedema.

The aim of this systematic literature review was to evaluate Ethiopian medicinal plants found to have antibacterial and antifungal properties *in vitro* studies.

In the context of this review, terms are defined as follows:

*“Ethiopian medicinal plants”* refer to plants that are found in Ethiopia and have been utilized traditionally for medicinal purposes by societies in Ethiopia and elsewhere.
*“In vitro anti-infective activity tests”* involve direct culture of the microorganisms in media and application of plant extracts to the media to evaluate their activity.
*“Anti-infective agents”* refer to agents (medicinal plant extracts, fractions, and/or compounds) that act against infective agents (bacteria, fungi and others) either by inhibiting the agent’s growth or by killing it.

## Materials and Methods

**FIGURE 1:**
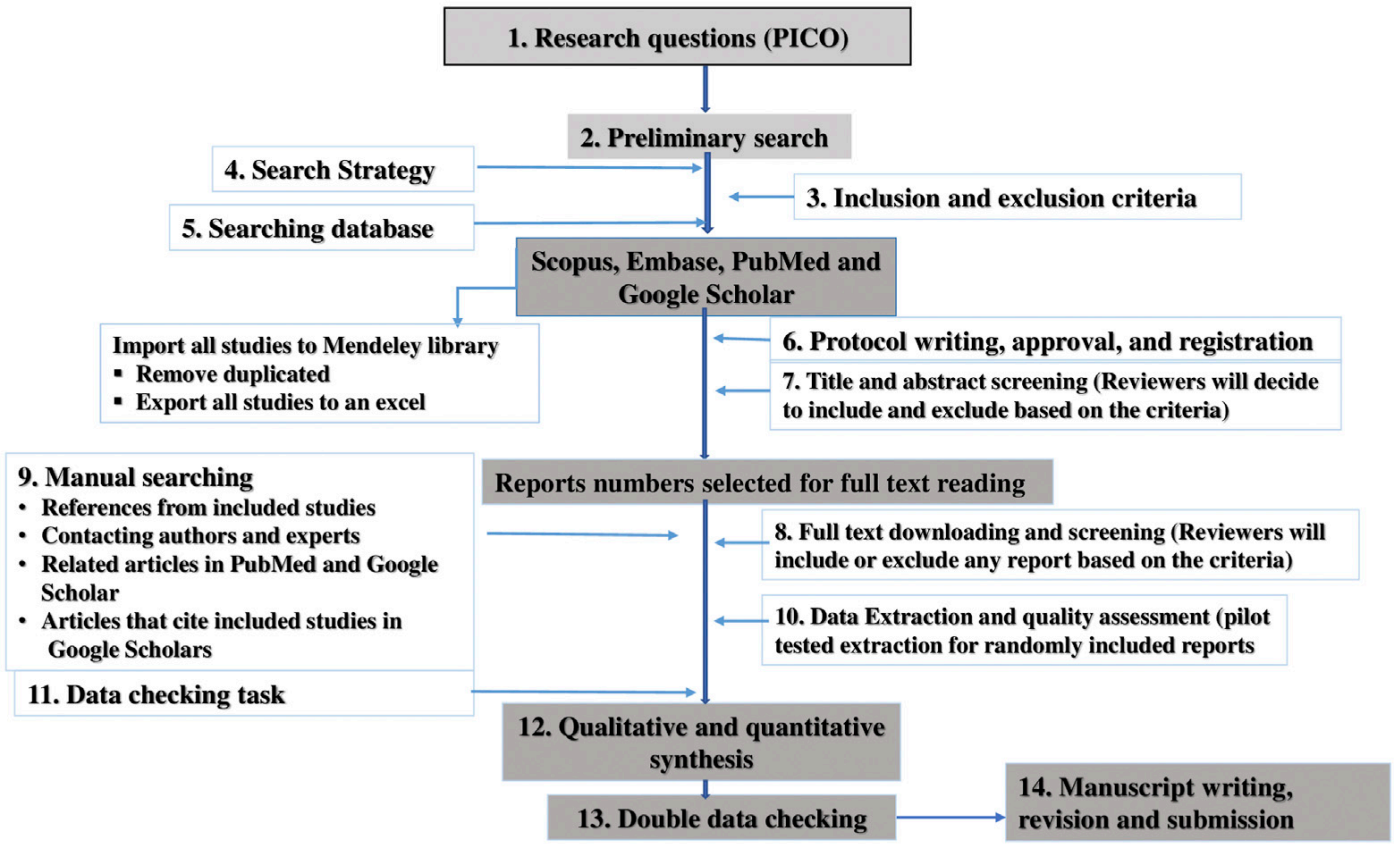
A step by step flow diagram to conduct the systematic.

To ensure inclusion of relevant information, the study was undertaken based on the guideline of Preferred Reporting Items for Systematic Reviews and Meta-analysis (PRISMA) (Lulekal et al., 2014) (Figure 1). The protocol for this review was registered on PROSPERO, with registration number CRD42019127471 (Nigussie et al., 2020a).

### Study Design

This systematic review considered all controlled *in vitro* antibacterial and antifungal studies the activities of Ethiopian medicinal plants. The components population, exposure (intervention), comparator and outcome (PICO) of this review are given underneath:

Study subjects: Microorganisms (bacteria and fungi) that were used for anti-infective efficacy tests of medicinal plants.

Intervention: Medicinal plants as whole plants or their adjuncts: seed, root, flower, bud and leaf extracts used in the experimental groups. The intervention products used were manufactured from a single or complex medicinal plant, plant extracts, or plant preparations, regardless of the types of the preparations (extracts, decoctions, tablets, capsules, pills, powders, injections or other types of preparations), but not synthesized compounds. There were no restrictions on dosage form, concentration, frequency of administration, dose, intensity or duration of medicinal plants used.

Comparator: Placebo (no intervention) or conventional (reference) drugs used for treatment of controls.

Outcomes: Outcomes were the rate of response to treatment, such as efficacy of medicinal plants in inhibiting or killing microbial growth (in culture media) in comparison with conventional drugs.

### Eligibility Criteria

Inclusion criteria: Published works including theses, articles and proceedings in English until June 2019 that deal with efficacy evaluation of antibacterial and antifungal activities *in vitro* studies.

Exclusion criteria: Newspapers, clinical trial studies and reviews.

### Information Sources

Electronic databases were looked at using a combination of free text keywords and Medical Subject Heading (MeSH) terms related to Ethiopian restorative plants investigated to have anti-inflammatory, anti-infective and wound healing activities. Scopus, Embase, PubMed/Medline and Google Scholar were utilized as sources of data for the search. Grey literature such as thesis, technical reports, working papers, evaluation reports, conference proceedings, patents, and preprints was too included in the review.

### Search Strategy

The search approach covered all articles containing descriptors available till June 28, 2019. Only articles composed in English have been used on this study. Structured search methods have been developed using the lexical terms of each database and targeting the “title” and “abstract” fields. We additionally searched manually using the references of already distributed works. The following search terms were used: Ethiopia, medicinal plants, Ethiopian medicinal plants, herbal products, care, management, therapeutic, lymphoedema, lymphedema, swelling, podoconiosis, elephantiasis, bacteria, anti-bacterial, fungi, anti-infective, antimicrobial, anti-fungal and different associated words or phrases.

### Selection of Studies

After electronic searching, the records were uploaded to Mendeley. Some studies were screened before being totally selected. All medicinal drug and antifungal studies were screened severally by two investigators (DN and TB by scanning the titles and abstracts of the articles that supported the inclusion criteria. For documents that fitted the inclusion criteria, the investigators scan the whole article to substantiate whether or not it met the criteria. Disagreements were resolved by discussion between the two investigators.

### Data Extraction and Management

Two reviewers (DN and TB) independently extracted data using a data extraction form. We performed a standardization exercise before beginning the review to make ensure consistency between the reviewers. The subsequent information were extracted: title, author, year of publication, statistical methods used, study duration, type of microorganism used for the study (clinical isolates or reference strains), concentrations of the plant extracts used for activity evaluation, reference drugs used, minimum inhibitory concentration (MIC) of extracts/fractions (µg/ml, mg/ml), zone of inhibition (mm), concentrations that inhibit microbial growth of the extracts/fractions, type of solvent extracts and fractions used for activity and safety, parts of plant used, extraction type, sources of the plants, place of collection, traditional use, scientific names of the plants, local names of the plants, voucher numbers, types and number of compounds isolated (if any). When individual studies had multiple treatments, groups were combined to avoid the possibility of introducing bias caused by multiple statistical comparisons with one control group (Lulekal et al., 2014).

### Outcomes Measured

For the *in vitro* studies of antibacterial and antifungal activities, the most outcomes measured were the percent of inhibition of growth of microorganisms, minimum inhibitory concentration, and concentration needed to inhibit multiplication of micro-organisms by 50% (IC_50_).

### Assessment of Risk of Bias

Two review authors (DN and TB) independently assessed the risk of bias for each included study. The internal and external validity of each study was evaluated using this tool. For the *in vitro* antibacterial and antifungal studies, a good practice for pharmaceutical microbiology laboratories guidelines (WHO) was used for quality assessment (WHO, 2009; World Heath Organisation, 2011). Criteria used to assess the quality of *in vitro* individual studies were reported in Nigussie et al. (2020a).

Then, the risk of bias criteria was judged as “low,” “high” or “unclear.” Studies with a low and medium risk of bias were considered for analysis, whereas high risk of bias studies were omitted from the analysis.

### Data Synthesis and Analysis

All studies included for data synthesis were classified into two different experimental models, i.e., antibacterial and antifungal activity studies, in keeping with the kind and purpose of the studies. Heterogeneity was evaluated descriptively from the narrative synthesised data, and potential reasons for heterogeneity were identified by examining individual study and subgroup characteristics. Interventional, methodological and statistical heterogeneity was apparent among the studies. Consequently, statistical pooling of studies for meta-analysis was not possible.

Instead, a narrative (qualitative) overview of the studies was conducted using textual descriptions of studies, grouping, and tabulation. Then, a detail of the characteristics of the studies compared the effect of each plant extract relative to controls, the main parameters measured/analyzed, the quality of included studies and the risk of bias of all studies was described.

## Results

### Literature Search Results and Description of Study Characteristics

In the search of Ethiopian medicinal plants used for their anti-inflammatory, wound healing or anti-infective activities, a total of 2,330 relevant articles were independently identified by two reviewers for preliminary review from electronic and manual searches. After removal of duplicates by reviewing relevant titles and abstracts, a total of 330 articles on antibacterial and antifungal activities were retrieved for full text review. After a detailed review of each article, 234 articles were excluded and a total of 96 articles were retained: anti-bacterial activity (*n* = 79) and anti-fungal activity (*n* = 17) (Figure 1 and Supplementary Tables S1–S3).

### Excluded Studies

In this review, we have identified that there were many studies conducted in these areas. However, most of the published articles did not meet the inclusion criteria due to i) Incomplete information: not reported the concentration of plant extracts used, the number of experimental duplicates, the method of outcome measurement, the negative and positive controls used, the sources and quality control of micro-organism, the time at which the outcomes were measured, the sources of the cell lines, and statistical methods used for data analysis; ii) Not relevant studies: clinical trials; newspaper reports; reviews, studies conducted on medicinal plants which are not growing in Ethiopia, activity was not conducted for human pathogens (animal and plant pathogens).

### Studies Included for the Antibacterial Activity of Medicinal Plants

#### Characteristics of the Studies

For the anti-bacterial studies, seventy-nine studies were eligible for data extraction. The year of publication ranged from 2003 to 2019. A total of 76 peer-reviewed full articles and 3 MSc thesis were included. The seventy-nine studies were conducted in Ethiopia (Seshathri and Thiyagarajan, 2011), India (eight), Kenya (seven), Iran (three), Sudan (three), Cameroon and South Africa (two each), China (one), Oman (one), Malaysia (one), Nigeria (one), Pakistan (one), Netherlands (one) and Tunisia (one) (Supplementary Tables S1, S3).

All the studies designs met the inclusion criteria and tests were performed according to the procedures described in the national, regional and international guidelines. The titles of the studies met the objectives stated in the studies. Of the 79 studies, 36 used agar well diffusion techniques with micro-dilution assay (MIC and MBC), and 28 used paper disc diffusion method with micro-dilution assay (MIC and MBC). Two of the studies used agar-well diffusion alone, while nine used microdilution methods for minimum inhibitory concentration and minimum bactericidal assays together with other methods, colorimetric assay and crystal violet assay methods.

A total of 144 plant species and four compounds were tested and all except two plant species were identified and authenticated by a botanist. Out of the 144 plant species 14 of them are found in Ethiopia (Table 1). Leaves were the most used plant parts for anti-bacterial tests (*n* = 82) (Table 1). All essential oils were extracted by steam distillation with a Clevenger-type apparatus, while maceration and Soxhlet techniques were the most frequently used techniques to extract plant materials.

**TABLE 1:** Summary of common medicinal plants identified from literature search as antibacterial.

S/N	Plant species	Family	Parts used	Number of citations	Ref
1	^a^ *Aloe trichosantha* A. Berger	Aloaceae	L latex	1	Oumer et al. (2014)
2	*Huernia hystrix* (Hook.f.) N. E. Br	Apocynaceae	S, R, W	1	Amoo et al. (2012)
3	^a^ *Entada abyssinica* Steud. ex A. Rich.	Fabaceae	Sb	1	Tchana et al. (2014)
4	*Entada africana* Guill. & Perr.	Fabaceae	Sb	2	Tchana et al. (2014), Kahaliw et al. (2017)
5	*Carica papaya* L*.*	Caricaceae	S	1	Akujobi et al. (2010)
6	*Persea americana* Mill.	Lauraceae	F	2	Tchana et al. (2014), Kahaliw et al. (2017)
7	*Croton macrostachyus* Hochst. ex Delile	Euphorbiaceae	L, Sb	3	Taye et al. (2011), Romha et al. (2018), Obey et al. (2016)
8	*Withania somnifera* (L.) Dunal	Solanaceae	L	3	Romha et al. (2018), Bisht and Rawat (2014), Rawat and Bisht (2014)
9	*Achyranthes aspera* L*.*	Amaranthaceae	L	3	Taye et al. (2011), Kalayou et al. (2012), Habtamu and Mekonnen (2017a)
10	*Brucea antidysenterica* J. F. Mill.	Simaroubaceae	R	1	Taye et al. (2011)
11	*Datura stramonium* L.	Solanaceae	L	3	Seshathri and Thiyagarajan (2011), Girmay (2015), Taye et al. (2011)
12	*Acokanthera schimperi* (A. DC.) Schweinf.	Apocynaceae	L	2	Taye et al. (2011), Tadeg et al. (2005)
13	*Phytolacca dodecandra* L’Hér.	Phytolaccaceae	R, F	2	Taye et al. (2011), Tadeg et al. (2005)
14	^a^ *Millettia ferruginea* (Hochst.) Hochst. ex Baker	Fabaceae	L	1	Taye et al. (2011)
15	*Solanum incanum* L*.*	Solanaceae	L	3	Taye et al. (2011), Mummed et al. (2018), Teka et al. (2015)
16	*Trachyspermum ammi (L.) Sprague* (L.) Sprague	Apiaceae	S	2	Omidpanah et al. (2016), Hassanshahian et al. (2014)
17	*Verbascum erianthum* Benth.	Scrophulariaceae	R, L	2	Tadeg et al. (2005), Yeabyo et al. (2018)
18	*Kosteletzkya begonifolia* (Ulbr.) Ulbr	Malvaceae	L	1	Tadesse et al. (2016)
19	*Leucas martinicensis* (Jacq.) R. Br.	Lamiaceae	L	1	Tadesse et al. (2016)
20	*Ranunculus multifidus* Forssk.	Ranunculaceae	L	1	Tadesse et al. (2016)
21	*Eugenia caryophyllata* Thunberg	Myrtaceae	L	1	Burt and Reinders (2003)
22	*Origanum vulgare* L.	Lamiaceae	L	1	Burt and Reinders (2003)
23	*Thymus vulgaris* L.	Lamiaceae	L	1	Burt and Reinders (2003)
24	*Nigella sativa* L.	Ranunculaceae	S	3	Chaieb et al. (2011), Kokoska et al. (2008), Mulat et al. (2015)
25	*Anthocleista schweinfurthii* Gilg	Gentianaceae	B, F & L	1	Djeussi et al. (2016)
26	*Caucalis melanantha* (Hochst.) Hiern	Apiaceae	B, F & L	1	Djeussi et al. (2016)
27	*Zehneria scabra (L. f.) Sond.*	Cucurbitaceae	W	1	Kongue et al. (2013)
28	*Combretum molle* R. Br. ex G. Don	Combretaceae	B	2	Asres et al. (2006), Regassa and Araya (2012)
29	*Rotheca myricoides* (Hochst.) Steane & Mabb*.*	Lamiaceae	L	1	Sileshi et al. (2007)
30	*Ficus palmata* Forssk*.*	Moraceae	L	1	Sileshi et al. (2007)
31	*Grewia ferruginea* A. Rich*.*	Tiliaceae	L	1	Sileshi et al. (2007)
32	*Periploca linearifolia Quart.-Dill.* & *A. Rich*	Asclepediaceae	L	1	Sileshi et al. (2007)
33	*Vernonia amygdalina (Syn: Gymnanthemum amygdalinum)* (Delile) Sch. Bip.	Asteraceae	F	5	Lulekal et al. (2014), Bacha et al. (2016), Mulat et al. (2015), Seshathri and Thiyagarajan (2011), Habtamu and Melaku (2018)
34	*Clematis hirsuta* Guill. & Perr*.*	Ranunculaceae	L	3	Geyid et al. (2005), Habtamu and Mekonnen (2017b), Moglad et al. (2014)
35	*Cuminum cyminum* L*.*	Umbellifers	L, S	2	Abdelraheim Belal (2017), Dua et al. (2013)
36	*Calpurnia aurea* (Aiton) Benth.	Fabaceae	L Ait, S, R	6	Romha et al. (2018), Tadeg et al. (2005), Adedapo et al. (2008), Kalayou et al. (2012), Ayal et al. (2019), Umer et al. (2013)
37	*Delonix elata* (L.) Gamble	Fabaceae	L	1	Vijayasanthi (2014)
38	*Spathodea campanulata* P. Beauv.	Bignoniaceae	L	1	Vijayasanthi (2014)
39	*Kalanchoe petitiana* A. Rich.	Crassulaceae	L	1	Tadeg et al. (2005)
40	*Lippia adoensis* Hochst	Verbenaceae	L	2	Tadeg et al. (2005), Teka et al. (2015)
41	*Olinia rochetiana* A. Juss*.*	Oliniaceae	L, SB	3	Tadeg et al. (2005), Teka et al. (2015), Yemata et al. (2019)
42	*Malva parviflora* L.	Malvaceae	R	2	Tadeg et al. (2005), Kalayou et al. (2012)
43	*Prunus africana* (Hook. f.) Kalkman	Rosaceae	Sb	1	Mwitari et al. (2013)
44	*Warburgia ugandensis* Sprague	Canellaceae	Sb	1	Mwitari et al. (2013)
45	*Plectranthus barbatus* Andrews	Lamiaceae	Sb	1	Mwitari et al. (2013)
46	*Embelia schimperi* Vatke	Myrsinaceae	Sb, S	2	Lulekal et al. (2014), Rondevaldova et al. (2015)
47	*Clausena anisata* (Willd.) Hook. f. ex Benth.	Rutaceae	St	1	Seshathri and Thiyagarajan (2011)
48	*Clematis simensis* Fresen.	Theaceae	St, L	2	Seshathri and Thiyagarajan (2011), Teshome and Teshale (2013)
49	*Rotheca myricoides* (Hochst.) Steane & Mabb*.*	Verbanaceae	St	1	Seshathri and Thiyagarajan (2011)
50	*Juniperus procera* Hochst. ex Endl.	Cuprassaceae	St	1	Seshathri and Thiyagarajan (2011)
51	*Justicia schimperiana* (Hochst. ex Nees)	Acanthaceae	St	1	Seshathri and Thiyagarajan (2011)
52	*Olea europaea* L.	Oleaceae	St	1	Seshathri and Thiyagarajan (2011)
53	*Phoenix reclinata* Jacq.	Arecaceae	St	1	Seshathri and Thiyagarajan (2011)
54	*Rubus apetalus* Poir.	Rosaceae	St	1	Seshathri and Thiyagarajan (2011)
55	*Sesbania sesban* (L.) Merr.	Leguminosae	St	1	Seshathri and Thiyagarajan (2011)
56	*Sida rhombifolia* L.	Malvaceae	St, W, R	3	Teka et al. (2015), Seshathri and Thiyagarajan (2011), Debalke et al. (2018)
57	*Spilanthes mauritiana* (Rich. ex Pers.) DC.	Asteraceae	F	1	Seshathri and Thiyagarajan (2011)
58	*Stereospermum kunthianum* Cham.	Bignoniaceae	St	1	Seshathri and Thiyagarajan (2011)
59	*Cymbopogon citratus* (DC.) Stapf	*Poaceae*	L	3	Ewansiha et al. (2012), Singh et al. (2011), Zulfa et al. (2016)
60	^a^ *Echinops kebericho* Mesfin	*Asteraceae*	R, Tu	2	Ameya et al. (2016), Belay et al. (2011)
61	*Costus speciosus (Koenig)* J. E. Smith	*Costaceae*	Rh	1	Duraipandiyan et al. (2012)
62	^a^ *Taverniera abyssinica* A. Rich.	*Fabaceae*	R	1	Gemechu et al. (2015)
63	^a^ *Bersama abyssinica* Fresen.	Melianthaceae	L, S, Sb	3	Teka et al. (2015), Geyid et al. (2005), Era and Nyanchoka (2016)
64	*Carissa spinarum* L.	Apocynaceae	R	1	Lulekal et al. (2014)
65	^a^ *Clutia abyssinica* Jaub. & Spach	Euphorbiaceae	R	1	Nwonuma et al. (2020)
66	*Cyathula cylindrica* Moq.	Amaranthaceae	R	1	Nwonuma et al. (2020)
67	*Dodonaea viscosa Jacq. subsp. angustifolia* (L. f.) J. G. West	Sapindaceae	L	1	Nwonuma et al. (2020)
68	*Jasminum abyssinicum* Hochst. ex DC*.*	Oleaceae	L	2	Lulekal et al. (2014), Goji et al. (2007)
69	*Maesa lanceolata* Forssk*.*	Primulaceae	L	1	Lulekal et al. (2014)
70	*Ocimum lamiifolium* Hochst. ex Benth.	Lamiaceae	L	2	Lulekal et al. (2014), Sahalie et al. (2018)
71	*Aframomum corrorima* (A. Braun) P. C. M. Jansen	Zingiberaceae	F	1	Bacha et al. (2016)
72	*Albizia schimperiana* Oliv.	Mimosaceae	R	1	Bacha et al. (2016)
73	*Curcuma longa* L.	Zingiberaceae	Rh	1	Bacha et al. (2016)
74	^a^ *Erythrina brucei Schweinf.* Emend. Gillett	Fabaceae	Sb	1	Bacha et al. (2016)
75	*Justicia schimperiana* (Nees) T. Anders.	Acanthaceae	S	1	Bacha et al. (2016)
76	*Ocimum gratissimum* subsp. gratissimum	Lamiaceae	L	1	Mulat et al. (2015)
77	*Ruta graveolens* L.	Rutaceae	L	1	Mulat et al. (2015)
78	*Premna resinosa* (Hochst.) Schauer	Lamiaceae	R	1	Njeru et al. (2015)
79	*Hagenia abyssinica* (Bruce) J. F. Gmel.	Rosaceae	L, Sb	1	Ngeny et al. (2013)
80	*Fuerstia africana* T. C. E. Fr*.*	Lamiaceae	Ae	1	Ngeny et al. (2013)
81	*Ekebergia capensis* Sparrm	Meliaceae	R	1	Ngeny et al. (2013)
82	*Asparagus racemosus* Willd.	Asparagaceae	Sb	1	Ngeny et al. (2013)
83	*Brassica nigra* (L.) W. D. J. Koch	Brassicaceae	S	2	Danlami (2016), Amare et al. (2013)
84	*Ocimum basilicum* L*.*	Lamiaceae	L	2	Sánchez et al. (2010), Cowan (1999)
85	*Syzygium aromaticum (L.) Merr.* & *L. M. Perry* (L.) Merr. & L. M. Perry	Lamiaceae	F	1	Burt and Reinders (2003)
86	*Elettaria cardamomum* (L.) Maton	Zingiberacea*e*	F	1	Kaushik et al. (2010)
87	*Cinnamomum verum* J. Presl	Lauraceae	Sb	1	Kwak et al. (2017)
88	*Kniphofia isoetifolia Hochst*	Asphodelacea	R	1	Meshesha et al. (2017)
89	*Blepharis cuspidata* Lindau	Acanthaceae	L	1	Gadisa et al. (2019)
90	*Boswellia ogadensis* Vollesen	Burseraceae	L	1	Gadisa et al. (2019)
91	*Thymus schimperi* Ronniger	Lamiaceae	L	3	Abreham et al. (2015), Nasir et al. (2015), Bekele (2015)
92	*Artemisia abyssinica* Sch.Bip. ex A. Rich*.*	Asteraceae	L	1	Belay et al. (2011)
93	*Satureja punctata* (Benth.) R. Br. ex Briq.	Lamiaceae	Ber	1	Zulfa et al. (2016)
94	*Schrebera alata* (Hochst.) Welw.	Oleaceae	B	1	Chalo (2015)
95	*Ormocarpum kirkii* S. Moore	Fabaceae	Ae	1	Chalo (2015)
96	*Cussonia holstii* Harms ex Engl.	Araliaceae	B	1	Chalo (2015)
97	*Helichrysum forskahlii* (J. F. Gmel.) Hilliard & B. L. Burtt	Asteraceae	W	1	Chalo (2015)
98	*Aloe macrocarpa* Tod.	Aloaceae	L	1	Desta et al. (2017)
99	*Moringa stenopetala* (Baker f.) Cufod.	Moringaceae	L	1	Hagos et al. (2018)
100	*Nicotiana tabacum* L.	Euphorbiaceae	L	2	Kalayou et al. (2012), Ameya et al. (2018)
101	*Zehneria scabra (L. f.) Sond.* (L. f.) Sond.	Curbitaceae	L	1	Abew et al. (2014)
102	*Ricinus communis* L*.*	Euphorbiaceae	L	1	Abew et al. (2014)
103	*Cissus quadrangularis* L*.*	Vitaceae	Ae	1	Mummed et al. (2018)
104	*Commelina benghalensis* L.	Commelinaceae	L	1	Tadeg et al. (2005)
105	*Euphorbia heterophylla* L.	Euphorbiaceae	R	1	Tadeg et al. (2005)
106	*Euphorbia prostrata* Aiton	Euphorbiaceae	W	1	Tadeg et al. (2005)
107	*Grewia villosa* Willd.	Malvaceae	L	1	Tadeg et al. (2005)
108	*Momordica foetida* Schumach.	Cucurbitaceae	F	1	Tadeg et al. (2005)
109	*Trianthema portulacastrum* L.	Aizoaceae	Ae	1	Tadeg et al. (2005)
110	*Schinus molle* L.	Anacardiaceae	L	1	Tadeg et al. (2005)
111	^a^ *Aloe sinana* Reynolds	Asphodelaceae	L	1	Minale et al. (2014)
112	*Maerua oblongifolia* (Forssk.) A. Rich*.*	Capparaceae	L, St	1	Minale et al. (2014)
113	^a^ *Clematis longicauda* Steud. ex A. Rich.	Ranunculaceae	L	1	Hawaze et al. (2012)
114	*Capsicum frutescens* L.	*Solanaceae*	F	1	Ameya et al. (2017)
115	*Apodytes dimidiata* E. Mey. ex Arn*.*	Icacinaceae	Sb	1	Teka et al. (2015)
116	*Asparagus africanus* Lam.	Asparagaceae	L	1	Mummed et al. (2018)
117	*Cucumis ficifolius* A. Rich*.*	Cucurbitaceae	R	1	Mummed et al. (2018)
118	*Gladiolus abyssinicus* Brongn. ex Lem.)	Iridaceae	Bu	1	Mummed et al. (2018)
119	*Guizotia schimperi* Sch. Bip.	Asteraceae	L	1	Mummed et al. (2018)
120	*Pavonia urens* Cav.	Malvaceae	L	1	Mummed et al. (2018)
121	*Premna schimperi* Engl*.*	Verbenaceae	L	1	Mummed et al. (2018)
122	*Pittosporum viridiflorum* Sims	Pittosporaceae	L	1	Mummed et al. (2018)
123	*Polygala sadebeckiana* Gürke	Polygalaceae	R	1	Mummed et al. (2018)
124	^a^ *Aloe harlana* Reynolds	Asphodelaceae	L	1	Asamenew et al. (2011)
125	*Ficus carica* L*.*	Moraceae	L	1	Kalayou et al. (2012)
126	*Solanum hastifolium* Hochst. ex Dunal	Solanaceae	L	1	Kalayou et al. (2012)
127	*Ziziphus spina-christi* (L.) Willd.	Rhamnaceae	L	1	Kalayou et al. (2012)
128	*Hibiscus micranthus* L. f.	Malvaceae	L	1	Begashaw et al. (2017)
129	*Linum usitatissimum* L.	Linaceae	S	1	Palla et al. (2015)
130	*Vernonia auriculifera* Hiern	Compositae	L	1	Albejo et al. (2015)
131	^a^ *Aloe pulcherrima* M. G. Gilbert & Sebsebe	Asphodelaceae	L	1	Abdissa et al. (2017)
132	*Lepidium sativum* L.	Cruciferae	S	1	Berehe and Boru (2014)
133	*Foeniculum vulgare* Mill.	Umbelliferae	L	1	Wodi (2016)
134	^a^ *Solanecio gigas* (*Vatke*) *C. Jeffrey*	Asteraceae	L	1	Goji et al. (2007)
135	*Lagenaria siceraria* (Molina) Standl.	Cucurbitaceae	L, S, F	1	Era and Nyanchoka (2016)
136	^a^ *Aloe trigonantha* L. C. Leach	Aloaceae	L	1	Megeressa et al. (2015)
137	*Pycnostachys eminii* Gürke	Laminaceae	L, S, R	1	Hussien et al. (2010)
138	*Moringa oleifera* Lam.	*Moringaceae*	S	1	Delelegn et al. (2018)
139	*Azadirachta indica A. Juss*A. Juss.	Meliaceae	L	3	Raja et al. (2013), Kavitha et al. (2017), Mohammed and Omer (2015)
140	*Pimenta racemosa* (Mill.) J. W. Moore	Myrtaceae	L	1	Burt and Reinders (2003)
141	*Nauclea latifolia* Sm	Rubiacea	B, F, L	1	Djeussi et al. (2016)
142	*Boehmeria virgata* var. *macrostachya* (Wight) Friis & Wilmot-Dear	Urticaceae	W	1	Djeussi et al. (2016)
143	*Erigeron floribundus* (Kunth) Sch. Bip.	Asteraceae	W	1	Djeussi et al. (2016)
144	*Zehneria scabra (L. f.) Sond.* (L. f.) Sond.	Cucurbitaceae	W	1	Djeussi et al. (2016)

Leaves = L, root = R, Stem Bark = SB, Fruits = F, Bark = B, Aerial = Ae, Flower = Fl, Stem = St, Rhizome = Rh, Bulbs = Bu, Seed = S, Berries = Be, Whole = W.

a Plant species endemic to Ethiopia.

A total of 25 gram-negative and 17 gram-positive bacteria were tested in the studies. Most of the microorganisms tested were American Type Culture Collection (ATCC) reference microorganisms and some were clinical isolates from samples. Among the gram-negative bacteria, *Escherichia coli* was tested against more than 70 types of medicinal plants, followed by *Pseudomonas aeruginosa, Klebsiella pneumoniae* and *Salmonella typhi* tested, which were tested against thirty-nine, twenty-eight and twenty-two medicinal plants, respectively. Among the gram-positives, *Staphylococcus aureus* was the most tested bacteria and was tested against sixty-six medicinal plants. Others included *Bacillus subtilis* (twelve), *Streptococcus pyogenes* (ten), and *Enterococcus faecalis* (eight) (Supplementary Table S1).

Twenty-six studies used SPSS statistical software for data analysis. One-way ANOVA was used to test the existence of statistically significant differences between mean zones of inhibition of controls and test substances. However, 45 studies did not report the statistical method used. The rest used the unpaired Student t-test to test the differences between treatment and control arms (Supplementary Table S1).

#### Main Parameters Analysed

For agar well and paper disc diffusion assay methods, the outcome measured at each test level was the diameter (mm) of the zone of inhibition of the control and experimental tests using a calibrated distance measuring instrument. The time of measurement for all included studies was after 24 h exposure to reference and test substances. For the microdilution methods (MIC and MBC), colour change for colorimetric assays or bacterial growth for non-colorimetric methods (visually identified as clear or turbid solution in test tubes after 24 h treatment) were used.

A wide range of concentrations and different types of units of measurements were used across the studies. Among the medicinal plants tested against the microorganisms, at least one plant constituent was active against bacteria. The units used to describe MIC were µg/ml, µl/ml, mg/ml, µg/disc, % (w/v) and ppm. Similarly, for the measurement of zone of inhibition of bacterial growth, millimetre (mm) and millimetre squared (mm^2^) were used.

The lowest concentration (8 µg/ml) of plant material that inhibited the growth of microorganisms was reported by (Chaieb et al., 2011). Eleven human pathogenic strains were tested against thymoquinone, a constituent of the black seed of *Nigella sativa* L*.* It was shown to inhibit the growth of *Bacillus cereus ATCC 14579* and *S. epidermidis CIP 106510 at* 8 µg/ml and *S. aureus ATCC 25923* 16 µg/ml (Chaieb et al., 2011). Ameya et al. (2017) reported the minimum inhibitory concentration of the alcoholic extract of *Echinops kebericho* Mesfin against *S. aureus, E. coli* and *E. faecalis,* which ranged from 3.12 to 12.5 µg/ml (Gemechu et al., 2015). Similarly, Oumer et al. reported the MIC values of latex of *Aloe trichosantha* Berger*,* which ranged from 10 to 100 µg/ml for bacterial species such as *bacillus* species, *E. coli* species, *Salmonella* species, *Shigella* species, *S. aureus* and *V. cholerae* (Ameya et al., 2017) (Supplementary Table S1).

Minale et al. reported the MIC values of *Aloe sinana* Reynolds and its compounds, Microdontn, Aloin and Aloinoside ranged from 10 to 50 µg/ml for the leaf latex, 10–25 µg/ml for Aloinoside, 5–200 µg/ml for Microdontn, 10–200 µg/ml for Aloin against gram-negative and gram-positive bacterial species. This was shown to have a strong anti-bacterial activity in comparison to the positive control, ciprofloxacin (Minale et al., 2014). Gadissa et al. tested the essential oils of *Blephari scuspidata*, *Boswellia ogadensis Vollesen* and *Thymus schimperi* Ronniger (Gadisa et al., 2019)*.* The MICs of *Blepharis cuspidate* against *S. aureus* (ACCT & MRSA), *E. coli* (MDR) and *K. pneumonia* (MDR) were 1.56, 12.5, and 3.12 µl/ml, respectively, which is comparable to ciprofloxacin activity (Gadisa et al., 2019).

Similarly*, Thymus schimperi* Ronniger was reported to have MICs of 3.12 µl/ml, 6.5 L/ml, and 3.12 µl/ml against *S. aureus* (ATCC & MRSA), *E. coli* (ACCT & MDR) and *K. pneumoniae* (ATCC & MDR), respectively, and the minimum bactericidal concentration (MBC) ranged from 3.12 to 12.5 µl/ml. In addition, *Boswellia ogadensis* Vollesen was shown to have MIC values of 3.12, 6.25, and 3.12 µl/ml against *S. aureus* (ATCC & MRSA), *E. coli* (ACCT & MDR) and *K. pneumoniae* (ATCC & MDR), respectively, and MBC ranged from 6.25 to 12.5 µl/ml (Gadisa et al., 2019).

The MIC values of *Boswellia ogadensis* Vollesen and *Thymus schimperi* Ronniger essential oil combination against *S. aureus* (ATCC & MRSA)*, E. coli* (*ACCT &* MDR), *K. pneumoniae* (*ATCC &* MDR) were 3.12, 6.25, and 1.56 µl/ml, respectively. The MBC ranged from 1.56 to 25 µl/ml. Similarly, the combination of essential oil of *T. Schimper* Ronniger and *Blepharis cuspidata* Lindau showed significant activity against *S. aureus* (ATCC *& MRSA*)*, E. coli* (*ACCT &* MDR) and *K. pneumoniae* (ATCC & MDR) with MIC of 0.39, 1.56, and 0.39 µl/ml, respectively, and with MBC values ranges from 0.39 to 3.12 µl/ml. The combined activities of essential oils of *B. cuspidata* Lindau and *B. ogadensis* Vollesen showed similar activity against *S. aureus* (ATCC & MRSA)*, E. coli* (ACCT & MDR) and *K. pneumoniae* (ATCC & MDR) with MICs of 1.56, 6.25, and 0.78 µl/ml, respectively. The MBC ranged from 1.56 to 25 µl/ml. These essential oils were shown to have comparable activity to ciprofloxacin (Gadisa et al., 2019).

Habbal et al. (2011) reported that 50% ethanol extracts of *Lawsonia inermis* L*.* demonstrated antibacterial activity against a wide range of gram-negative and positive bacterial strains with the highest antibacterial activity against *P. aeruginosa*. Nagarajan et al. (2013) reported that ethanol, chloroform, hexane and methane extracts of *L. inermis* L. showed nearly equal zones of inhibition against *B. subtitles, S. aurous, and E. coli* at 400 mg/kg comparable to that of tetracycline. Ethanol, methanol, and ethyl acetate extracts of *Azadiractha indica* A. Juss were reported by Maleki et al. to have a wider zone of inhibition against *P. aeruginosa, S. aureus* and *E. faecalis* at 300 mg/ml. The extracts had bactericidal activity against both reference and clinical isolates of *S. aureus* and *P. aeruginosa*, and bacteriostatic activity against *E. faecalis* (Maleki et al., 2018).

The degree of bacterial growth inhibition, as determined by values of diameter of inhibition zone (IZ) of the respective plant extracts, varied among the extracts and microorganisms. The widest inhibition was reported by Bacha et al. (2016) who showed the inhibitory zones of petroleum ether extract (500 mg/ml) of seeds of *Nigella sativa L.* to be 44 ± 0.31 mm against *Bacillus cereus* and 40 ± 2.33 mm *against B. cereus* ATCC 10987 compared to that of gentamycin (29 mm). Wide zones of inhibition were recorded for the petroleum ether extract of stem of *Kosteletzkya begonifolia* Ulbr. and stem of *Leucas marthineensis* (Jacq.) R. Br. against *E. coli*, *S. typhimurium*, *S. aureus* and *P. aeruginosa* at all concentrations, comparable to ciprofloxacin (Tadesse et al., 2016). In another study, acetone extract of *Capsicum frutescens L.* against ATCC *S. aureus* at a concentration of 0.1 mg/ml was reported to produce an inhibitory zone of 28 mm (Ameya et al., 2017).

#### Quality of Included Studies (Bias Analysis)

Critical appraisal of the studies included was done using the checklist for Good *In Vitro* Method Practices (OECD) and the WHO Good Practice for Microbiology Laboratory. Seven main criteria were used to evaluate the validity of methodological and reporting qualities (details are in the *Materials and Methods* section) (Supplementary Table S1).

Under the main checklist there were thirty criteria to evaluate the internal validity of the studies. Studies with unacceptable levels of bias were excluded. However, the studies included still had some weaknesses in reporting the status of microbiology facilities, regular equipment, apparatus maintenance and calibration. In addition, there was lack of clarity as to whether the test methods were validated or not; and there was also lack of evidence as to whether the microbiological tests were performed and supervised by an experienced person qualified in microbiology or equivalent, and whether the opinions and interpretations of test results in reports were done by authorized personnel with suitable experience and relevant knowledge.

There was also some methodological weakness. For instance, the number of replicates for each testing condition, including concentration level(s) used for the reference and control item(s), and test items were not specified in some studies. None of the studies reported the applicability domain of the *in vitro* methods or any limitations or exceptions to the methods. Four studies did not report complete information about the degree of inhibition of bacterial growth and the concentrations by the respective medicinal plants, and one study did not mention the unit of measurement of the zone of inhibition by plant extracts.

We categorized the judgment of bias as “yes,” “no” or “unclear.” A “yes” judgement indicated a low risk of bias; and a “no” judgment indicated high risk of bias; the judgment was “unclear” if insufficient details were reported to assess the risk of bias properly.

### Studies Included on Anti-fungal Activity of Medicinal Plants

#### Characteristics of the Studies

Seventeen studies that evaluated anti-fungal activities of Ethiopian medicinal plants were included. The year of publication of the studies included ranged from 2000 to 2018, and studies were conducted in six different countries, Ethiopia (*n* = 7), Kenya (*n* = 1), India (*n* = 5), Colombia (*n* = 1), Lithuanian (*n* = 1), Republic of Korea (*n* = 1), and Romania (*n* = 1). Sixteen studies were peer reviewed full articles and one was an MSc thesis. Five studies used microdilution assay, two used agar well diffusion method, and twelve used both methods (Supplementary Table S2).

Medicinal plants claimed to have anti-fungal activities were tested against different fungal species and one species of yeast. These were *Candida albicans*, *Aspergillus* species, *Trichophyton* species, *Microscopium* species, *Penicillium* species, *Fusarium* species, *Epidermophyto*n species and *Rhodotorula rubra.* Aspergillus species (*n* = 18) were the most-studied fungi, followed by *Trichophyton* species (*n* = 13) and *Candida albicans* (*n* = 10).

A total of 42 different species of medicinal plants were tested against different fungi, and all of them identified and authenticated by botanists and with voucher numbers. Out of these, four plant species are endemic to Ethiopia (Table 2).

**TABLE 2:** Summary of common medicinal plants identified from literature search for anti-fungal activity.

S/N	Plant species	Family	Parts used	Number of citations	References
1	*Combretum molle* R. Br. ex G. Don	Combretaceae	Sb	1	Asres et al. (2006)
2	*Clerodendrum myricoides* R. Br.	Laminaceae	L	1	Sileshi et al. (2007)
3	*Ficus palmata* Forssk*.*	Moraceae	L	1	Sileshi et al. (2007)
4	*Grewia ferruginea* A. Rich.	Tiliaceae	L	1	Sileshi et al. (2007)
5	*Periploca linearifolia* Quart.-Dill. and A. Rich.	Asclepeiaceace	Ae	1	Sileshi et al. (2007)
6	*Allium sativum* L.	Liliaceae	C	1	Vaijayanthimala et al. (2000)
7	*Allium schoenoprasum* L.	Liliaceae	C	1	Mohammed and Omer (2015)
8	*Allium cepa* L.	Liliaceae	Bu	1	Mohammed and Omer (2015)
9	*Acalypha indica* L*.*	Meliaceae	L	1	Mohammed and Omer (2015)
10	*Azadirachta indica* A. Juss	Euphorbiaceae	L	3	Viswanathan and Jayachandra (2017), Salazar et al. (2015), Iván et al. (2015)
11	*Camellia sinensis* (L.) Kuntze	Theaceae	L	1	Vaijayanthimala et al. (2000)
12	*Senna alata* (L.) Roxb*.*	Caesalpiniaceae	L	1	Mohammed and Omer (2015)
13	*Cassia fistula* L.	Caesalpiniaceae	L	1	Mohammed and Omer (2015)
14	*Senna occidentalis* (L.) Link	Caesalpiniaceae	L	1	Mohammed and Omer (2015)
15	*Coffea arabica* L.	Rubiaceae	S	1	Mohammed and Omer (2015)
16	*Curcuma longa* L.	Zingiberaceae	R	1	Mohammed and Omer (2015)
17	*Lawsonia inermis* L*.*	Lythraceae	L	3	Nabila et al. (2018), Vaijayanthimala et al. (2000), Rahmoun et al. (2013)
18	*Murraya koenigii* (L.)	Rutaceae	L	1	Mohammed and Omer (2015)
19	*Ocimum tenuiflorum* L.	Labiatae	L	1	Mohammed and Omer (2015)
20	*Piper betle* L.	Piperaceae	L	1	Mohammed and Omer (2015)
21	*Cullen corylifolium* (L.) Medik*.*	Papilionaceae	S	1	Mohammed and Omer (2015)
22	*Cinnamomum porrectum* (Roxb.) Kosterm*.*	Lauraceae	S	1	Bora (2016)
23	*Phyla nodiflora* (L.) Greene	Verbenaceae	L	1	Bora (2016)
24	*Cestrum nocturnum* L.	Solanaceae	F	1	Bora (2016)
25	*Trachyspermum ammi* (L.) Sprague	Apiaceae	S, F	2	Gemeda et al. (2014), Sharifzadeh et al. (2015)
26	*Leptospermum petersonii* F. M. Bailey	Myrtacea	S	1	Park et al. (2007)
27	*Syzygium aromaticum* (L.) Merr. and L. M. Perry	Myrtacea	S	2	Park et al. (2007), Rana et al. (2011)
28	^a^ *Echinops kebericho* Mesfin	Asteraceae	R	1	Ameya et al. (2016)
29	^a^ *Taverniera abyssinica* A.Rich.	Fabaceae	R	1	Ameya et al. (2016)
30	*Cymbopogon martini* (Roxb.) W.Watson	Poaceae	Ae	1	Gemeda et al. (2014)
31	*Foeniculum vulgare* Mill.	Apiaceae	Ae	1	Gemeda et al. (2014)
33	*Dodonaea viscosa* Jacq.	Sapindaceae	L	1	Duraipandiyan and Ignacimuthu (2011)
34	*Rumex nervosus* Vahl	Polygonaceae	R	1	Duraipandiyan and Ignacimuthu (2011)
35	*Rumex abyssinicus* Jacq.	Lamiaceae	R	1	Duraipandiyan and Ignacimuthu (2011)
36	*Thymus vulgaris L (Oleogel)*	Lamiaceae	L	2	Jain and Sharma (2017), Tadele et al. (2008)
37	*Juniperus communis* L.	Cupressaceae	F	1	Fierascu et al. (2018)
38	*Bersama abyssinica* Fresen	Francoaceae	Sb	1	Teka et al. (2015)
39	^a^ *Inula confertiflora* A. Rich.	Compositae	L, F	1	Messele (2004)
40	*Clematis simensis* Fresen.	Ranunculaceae	L	1	Fierascu et al. (2018)
41	*Zehneria scabra* (L. f.) Sond.	Cucurbitacea	L	1	Fierascu et al. (2018)
42	^a^ *Pycnostachys abyssinica* Fresen.	Labiatae	L	1	Fierascu et al. (2018)

Leaves = L, root = R, Stem Bark = SB, Fruits = F, Bark = B, Aerial = Ae, Flower = Fl, Stem = St, Rhizome = Rh, Bulbs = Bu, Seed = S, Berries = Be, Whole = W, Clove = C.

a Plant species endemic to Ethiopia.

Leaves (*n* = 21), seeds (*n* = 6), roots (*n* = 4), stem bark (*n* = 2), aerial parts (*n* = 2), cloves (*n* = 2), bulbs (*n* = 1), fruits (*n* = 4), were the plant parts used (Table 2). The maceration technique was the most frequently used method of extraction for plant extracts, followed by Soxhlet. Steam distillation with Clevenger-type apparatus was used for extraction of essential oils. Hydro-alcohol solvents were the most frequently used solvents for the extraction of plant materials followed by aqueous solvents.

Six studies used the agar well diffusion (AWD) method, six the micro dilution (MID) method and five both methods. For both experimental methods, the duration of exposure of the microorganisms to the extracts ranged from 2 to 7 days incubation time; and outcomes were measured after this. Zone of inhibition of fungal growth, turbidity (visually) and anti-fungal activity index (%) were the outcomes measured in the included studies. All the measurements were replicated three times and the results were presented as mean ± SD. One-way ANOVA followed by Tukey’s test was used to compare extraction solvents and the difference in the sensitivity of the test microorganisms.

#### Main Parameters

The antifungal activity of plant extracts was measured in a similar way as that of the anti-bacterial activity. These were zone of inhibition of fungal growth for the agar well and paper disc diffusion methods and fungal growth which distinguished clear and turbid solutions for the micro-dilution methods, measured after incubation periods.

A wide range of concentrations and units of measurement were used across the studies. For the MIC and minimum fungicidal concentration (MFC) mg/ml, µg/ml and activity index in percent (%), and mm was used for the measurements of ZI in AWD assays.

The activity index of the extracts was determined using the following formula:

Ameya et al. (2016) reported that the methanol extract of *Echinops kebericho* Mesfin against *Aspergillus flavus* and *Candida albicans* had MICs of 6.25 and 3.12 µg/ml, respectively; and the MFC of methanol extract to be 12.5 and 6.25 µg/ml against *A. flavus* and *C. Albicans,* respectively. The ethanol extract had MICs of 12.5 and 6.25 µg/ml against *A. flavus* and *C. albicans,* respectively with fungicidal activity of 22.92 and 12.50 µg/ml, respectively. The zone of inhibition of the methanol extract against *C. albicans* and *A. flavus* were 18.66 ± 0.57 and 20.33 ± 0.57 mm, respectively. In this study, ethanol and methanol extracts of *E. kebericho* Mesfin were shown to have comparable activity with ketoconazole (Supplementary Table S2).

Kasparaviciene et al. evaluated the activity of oleo-gels formulated with different concentrations of thyme essential oil. The MIC value of 0.25% essential oil of thyme in oleo-gels against *C. albicans* was 0.05% (Kasparaviciene et al., 2018). In another study, the antifungal activity of *T. vulgaris* essential oil against dermatophytic fungi was reported by Neetu et al. to have a very strong antifungal activity at low concentrations. The MIC values ranged from 0.05 to 0.1 µl/ml; and the MFC ranged from 0.05 to 2 µg/ml against the dermatophtic fungi (Jain and Sharma, 2017) (Supplementary Tables S2, S3).

The seed extracts of *Trachyspermum ammi* (L.) Sprague (0.2 mg/ml) and the leaf extract of *Cestrum nocturnum L.* (0.2 mg/ml) exhibited the widest zones of inhibition, at 38.3 and 31.3 mm, respectively against *C. albicans*. Similarly, the methanol extract of *E. kebericho* Mesfin exhibited ZI of 20.33 ± 0.57 mm against *C. albicans* and 18.6 mm against *A. flavus* (Supplementary Table S2)*.* Salazar *et al* showed that leaf and seed oil extracts of neem tree inhibited the growth of *Trichophyton menta, Trichophyton rubrum, Epidermophyton floccos* and *Microsporumcanis*. Whereas Simhadri *et al* reported that the aqueous extract of *Azadirachta indica* A. Juss*.* leaves had superior activity against *T. rubrum, M. gypseum*, *E. floccosum*, and *Candida* species (Viswanathan and Jayachandra, 2017).

#### Quality of Included Studies (Bias Analysis)

Checklists employed for antibacterial studies were also used in the antifungal studies. There were gaps in methodology as well as in reporting and interpreting the outcomes. Validation of the test methods before conducting the experiments was not reported for all included studies, and eight studies did not report the statistical methods used.

There was no evidence that the anti-fungal activity tests were performed or supervised by an experienced person qualified in microbiology or equivalent. Similarly, there was no report on whether the microbiology facilities were fit for purpose or detailed description of the workflow for the microbiology methods and related processes. Furthermore, nine studies did not report the statistical methods used, not expressed an estimate of the uncertainty of the test result on the test report, and limitations of the test were not reported clearly.

## Discussion

The purpose of this review was to demonstrate the activities of Ethiopian medicinal plants as antimicrobial agents that might potentially be used for limb care (particularly, of tropical lymphoedema and associated wounds). This section discusses the efficacy of plant extracts and their secondary metabolites investigated as antibacterial and antifungal, and the most frequently used models.

This systematic review identified a total of 96 articles covering two different experimental models, i.e., 79 antibacterial activity and 17 antifungal activity models. Overall, medicinal plant extracts tested for these two conditions in *in vitro* were shown to have good activity. Despite the heterogeneity of the studies, all plant extracts investigated succeeded in inhibiting bacterial and fungal growth.

In this review of antibacterial activity, a total of 144 medicinal plant species and four compounds were investigated against 25 gram-negative and 17 gram-positive bacteria using agar well diffusion, paper disc diffusion, broth micro/macro-dilution and agar dilution method. A summary of plant species whose extracts and their isolated compounds were shown to have significant *in vitro* activity against bacteria is the focus for our discussion.

Chaieb et al. reported the MIC of thymoquinone, constituent of *N. sativa* L., which was shown to have MIC of 32 µg/ml against *V. parahaemolyticus ATCC 17802* and *E. faecalis* ATCC 29212, 16 µg/ml against *L. monocytogene ATCC 19115*, 8 µg/ml against *B. cereus ATCC 14579, S epidermidis CIP 106510, M. luteus NCIMB 8166, S. aureus ATCC 25923* and *S. epidermidis CIP 106510* in a broth microdilution assay method. Its activity was shown to be similar to the standard drugs gentamycin and erythromycin (Chaieb et al., 2011). This finding agrees with the report of Kokoska et al., who tested the essential oil of *N. sativa* L. seed against gram-positive bacteria. Thymoquinone, the main constituent of the essential oil, was shown to have a potent bacteriostatic effect with MIC ranging from 8 to 64 µg/ml in broth microdilution method (Kokoska et al., 2008). However, *E. coli ATCC 35218*, *S. enterica serovar Typhimurium ATCC 14028*, and *P. aeruginosa ATCC27853* were found to be resistant to this compound (MIC >512 µg/ml) (Kokoska et al., 2008).

Ameya et al. (2016) tested the alcoholic extract of *E. kebericho* Mesfin against *S. aureus, E. coli and E. faecalis* and demonstrated significant activity with MIC ranged from 3.12 to 12.5 µg/ml using AWD, while *E. coli* was found to be resistant. Anwar et al. assessed the antimicrobial activity of latex of *Aloe trichosantha* A. Berger and its compounds (aloin A/B and aloin-6′-O- acetate A/B), which were effective against *E. coli, Salmonella* and *V. cholerae* strains with an average MIC value of 25 µg/ml. The activities of the test substances could be due to changes to cell wall integrity, enzymatic activity and protein inactivation in the microorganisms (Oumer et al., 2014).

Minale et al. performed anti-bacterial activity tests on *Aloe sinana* Reynolds and its compounds (Microdontin, Aloin and Aloinoside) against 21 strains of bacteria using the disk diffusion method. The leaf latex showed high inhibitory activities against *B. pumillus 82, B. subtilis ATCC 6633 and S. aureus ML 267, E. coli K99, E. coli K88, E. coli CD/99/1, E. coli LT37, E. coli 306, E. coli 872, E. coli ROW 7/12, E. coli 3:37C, S. enterica TD 01, S. typhi Ty2, S. boydii D13629, S. dysentery 8, S. flexneri Type 6, S. soneii 1, V. cholerae 85, V. cholerae 293, V. cholerae 1,313 and V. cholerae 1,315* at a concentration of 200 µg/ml, which showed comparable activity to the standard drug ciprofloxacin. Similarly, compounds isolated from *Aloe sinana* Reynolds were shown to have high activity against *E. coli, S. typhi Typ 2, Shigella, S. aureus and V. cholerae*, comparable to the reference drug, ciprofloxacin (Minale et al., 2014). The leaf latex’s action was due to the secondary metabolite anthraquinones, which possess a range of functional groups and have the ability to disrupt bacterial cell wall permeability and inhibit nucleic acid synthesis and then cause death of the microorganism (Malmir et al., 2017; Xu et al., 2018).

According to Gadisa et al., combined essential oils of oregano-basil, basil-bergamot, oregano-bergamot and oregano-perilla have significant activity against *S. aureus, E. coli, B. subtilis and S. cerevisiae,* respectively. The synergistic effect of these essential oils may be due to synergistic or additive interactions between different classes of compounds such as phenols, aldehydes, ketones, alcohols, esters, ethers or hydrocarbons, which might act on the same target or different targets (Gadisa et al., 2019). This finding is consistent with Nasir et al. (Haroun and Al-Kayali, 2016), who postulated that the ability of plant extracts to act synergistically with antibiotics and other plant extracts could be considered a new approach to combat antimicrobial resistance. There is low risk of bacterial resistance in plant extracts and antibiotics combinations, due to the varied modes of action of the compounds present in the extracts, to which the organism had never been exposed before (Haroun and Al-Kayali, 2016).

Antibacterial activity of *Lawsonia inermis L.* was also reported against wide range of gram-positives and gram-negatives (Annavarapu et al., 2016; Nabila et al., 2018). This could be due to the presence of a compound, 2-hydroxy-1, 4-naphthoquinone. Quinones are the main constituent in the leaves of *L. Inermis* L. and are known in making complexing irreversibly with nucleophilic amino acids, leading to inactivation of the protein and loss of function in microorganisms. Cell wall adhesions, polypeptides and membrane bound enzymes are the targets in microbial cells (Nabila et al., 2018).

In another anti-microbial study, the leaf and stem bark extracts of *Azadirachta indica* A. Juss. exhibited significant antibacterial activity against a wide range of bacteria due to the tricyclic diterpenoids isolated from stem bark, and azadirachtins, quercetin and β-sitosterol isolated from the leaves (Raja et al., 2013).

Bacha *et al* tested 18 plant extracts against *E. coli K12 DMS 498, S. aureus DMS 346, B. cereus ATCC 10987, B. cereus, Lab strain* and *P. aeruginosa 1,117* using AWD and MID methods. The highest ZI was recorded with petroleum ether extract of *N. Sativa* L against *B. cereus* and *B. cereus ATCC 10987;* and mature unripe fruit oil of *Aframomum corrorima (A. Braun)* P. C. M. Jansen against *S. aureus.* The activities of petroleum ether extract of seed of *N. sativa* L against both laboratory isolated and reference strain of *B. cereus* were greater than the activity of gentamycin sulphate. The oil extract of unripe fruit of *A. corrorima* (A. Braun) P. C. M. Jansen was shown to have an activity comparable to the reference drug gentamycin sulphate. *P. aeruginosa* was the most resistant to all the plant extracts tested in this study (Bacha et al., 2016). The antimicrobial activities of extracts of *A. corrorima*, *Nigella sativa* L*., A. angustifolium* (Sonn.) K. Schum. and *V. amygdalina* (Delile) Sch. Bip. were due to the presence of phenol, tannin, saponin and flavonoids, flavonoids and terpenoids compounds and their combinations (Bacha et al., 2016). The antibacterial activity of flavonoids is well documented and found in almost all parts of the plants, which inhibit the energy metabolism and synthesis of nucleic acids of different microorganisms (Cushnie and Lamb, 2005). Furthermore, tannins were reported to have antibacterial activity against *S. aureus,* acting by inducing complexation with enzyme or substrates and causes toxicity; and altering the membrane of the microbes (Akiyama, 2001).

Many studies have been carried out to screen medicinal plants for their antifungal activity, and various groups of researchers have initiated antifungal programs for traditionally used plants. Classes of compounds from plant metabolites, such as terpenoids (isoprenoids), saponins, phenolic compounds, flavonoids, coumarins, alkaloids, proteins and peptides showed anti-fungal activity against different fungal species (Aqil et al., 2010; Duraipandiyan and Ignacimuthu, 2011). Under this review, 15 studies were included comprising 42 species of plant extracts against 50 species of fungus using agar well diffusion, disc diffusion, macro/microdilution and agar dilution methods.

Alcoholic extracts (methanol and ethanol) of *E. kebericho* Mesfin were tested by Ameya et al. against *A. flavus* and *C. albicans* using disc diffusion and agar dilution methods, and shown to cause significant inhibition at low concentration, comparable to the reference drug ketoconazole. The alcoholic solvents have the ability to extract phenolic compounds such as flavonoids, anthocyanins and phenolic acids which may contribute to the antifungal activity of the extracts (Gemechu et al., 2015).

Kasparaviciene et al. tested the activity of oleo-gels, formulated with different concentrations of thyme essential oil against *C. albicans* by broth dilution method, which showed significant activity with MIC value of 0.25%. Thymol was reported the major constituent of the thyme essential oil in this study. The biological activity of thyme essential oil depends on its yield and chemical composition, and the essential oils have several chemical names depending on the main constituents they have, such as thymol, carvacrol, terpineol, and linalool (Kasparaviciene et al., 2018).

Similarly, Jain et al. reported the antifungal activity of *T. vulgaris* essential oil against *T. mentagrophytes* MTCC 7687*, M. gypseum* MTCC 452, *M. fulvum*MTCC2837*, T. rubrum* MTCC 296, *T. soudanense* (isolate) and *T. interdigitale* (isolate) using macro-dilution method. *T. vulgaris* L. essential oil was shown to have significant activity against the dermatophytes with MIC ranges from 0.02 to 0.1 μl/ml (Jain and Sharma, 2017). These activities could be due to high content of phenolic compounds and potent vapour activity against dermatophytes (Soković et al., 2009). This finding agrees with the report of Marina et al., which showed the activity of *T. vulgaris* L. essential oil against *Alternaria alternata*, *Fusarium tricinctum*, all *Aspergillus* species and dermatomycetes at concentration of 0.25 µL/ml and *Phomopsis helianthi* and *Cladosporium cladosporioides* at 0.125 µl/ml by macro-dilution method (Soković et al., 2009).

In another study, *T. ammi* (L.) Sprague seed extract exhibited potent antifungal efficacy, with a maximum MZ of 38.3 mm diameter against *C. albicans* using the AWD method (Gemeda et al., 2014). This is in agreement with the finding of Sharifzadeh et al., which evaluated *T ammi* (L.) Sprague essential oil against *C. albicans*, which were fluconazole-resistant, with MIC values ranging from 300 to 400 µg/ml (Sharifzadeh et al., 2015). The extracts of *A. indica* was also shown to have antifungal activity, attributable to the terpenoids. The fractions of *A. indica* A. Juss have complex mixtures of compounds reported to have synergistic and additive effect of against fungus (Salazar et al., 2015).

## Conclusion

The present study showed that plant extracts and compounds traditionally used in Ethiopia are promising anti-infective agents. In this review *Calpurnia aurea* (Aiton) Benth., *Croton macrostachyus* Hochst. ex Delile*, Withania somnifera* (L.) Dunal*Achyranthes aspera* L., *Datura stramonium* L., *Solanum incanum* L., *Verbascum erianthum* Benth., *Nigella sativa* L., *Gymnanthemum amygdalinum* (Delile) Sch. Bip., *Olinia rochetiana* A. Juss., *Sida rhombifolia* L*, Bersama abyssinica* Fresen. and *Azadirachta indica* A. Juss are the most studied plants species against bacteria, and *Azadirachta indica* A. Juss and *Lawsonia inerms* L. against fungal species. Thymoquinone, a constituent of the black seed of *Nigella sativa* L., alcoholic extract of *Echinop skebericho* Mesfin, *Aloe sinana* Reynolds and its compounds (Microdontin, Aloin and Aloinoside)*,* alcoholic extract of *Azadiractha indica* A. Juss and *Lawsonia inerms* L. are the most effective plant materials against gram negative and gram-positive species. In addition, *Azadiractha indica* A. Juss and *Lawsonia inerms* L*.* have activity against a wide range of gram-negative and positive bacterial strains. Similarly, methanol extract of *Echinops kebericho* Mesfin and oleo-gels formulated with different concentrations of thyme essential oil are the most effective against different fungal species.

### Strength and Limitations

This systematic review will provide up-to-date information on Ethiopian medicinal plants used as anti-infective agents that might potentially be used for limb care (lymphoedema and associated wounds). This information could lead to the development of more research on the investigation of the effect of medicinal plants on against infection for future therapeutic use. However, as it will summarizes studies written only in English this could be and which is considered the anticipated one of the limitations of this review. In addition, this study has will considered a wide range of methodological approaches and used different.

### Implications for Future Research and Recommendations

It is vital to systematically summarize, and document medicinal plants tested against different disease agents and used traditionally for treatment, and to test further their effectiveness against a range of disease-related pathology such as wounds in patients with lymphoedema. Information about many medicinal plants is fragmented, meaning that systematic compilation and synthesis is important. The findings of this systematic review helped us in identifying and prioritizing medicinal plants species for further investigation to determine their efficacy as alternative therapy to microbial infections associated with lymphoedema.

## Data Availability

The original contributions presented in the study are included in the article/Supplementary Material, further inquiries can be directed to the corresponding author.
