# Supplementary material for: Antibacterial and Antifungal Activities of Ethiopian Medicinal Plants: A Systematic Review
Source: Front Pharmacol. 2021 Jun 1;12:633921. doi: 10.3389/fphar.2021.633921 (PMC8203926; doi:10.3389/fphar.2021.633921)
Supplement: Supplementary file 1 [file Table1.docx]

**Supplementary table 1. List of included studies for antibacterial activity studies**

| **S/N** | **Authors** | **Name of the assay (method)** | **Name of microorganism (references & isolates) tested against medicinal plants activity conducted** | **Scientific name of the plant(s)/ compounds with parts used** | **Family of the plants** | **Types of plants extracts/fractions /compounds used for activity** | **Outcome measured *** |
| --- | --- | --- | --- | --- | --- | --- | --- |
| 1 | Oumer *et al,* 2014 | DD | *Bacillus pumillus 82,*  *B*. *Subtilis ATCC 6633,*  *S. aureus ML 267,*  *E. coli (K99, K88, CD/99/1, LT37, 306, 872, 7/12, 3:37C),*  *Salmonella enteric TD 01, S. typhi Ty2,*  *Shigella boydii D13629,*  *S. dysentery 8,*  *S. flexneri Type 6,*  *S. soneii 1,*  *Vibrio cholerae (293, 1313 and 1315)* | *Aloe trichosantha* A.Berger (L) | Aloaceae | Leaf latex; compounds (Aloin A/B (1), Aloin-6'-O-acetae A/B (2) | ZI |
| 2 | Amoo *et al*, 2012 | MiD bioassay | *Bacillus subtilis ATCC 6051*  *E. coli ATCC 11775 and*  *K. pneumoniae ATCC 13883*  *S. aureus ATCC 12600;* | *Huernia hystrix* (Hook.f.) N.E.Br. (W) | Apocynaceae | Petroleum ether, dichloromethane and  80% ethanol | MIC & MBC |
| 3 | Techana *et al*, 2012 | MiD (colorimetric) assay | *E. coli (ATCC8739, AG100A),*  *E. aerogenes (ATCC13048, CM64),*  *K. pneumoniae (ATCC11296, Kp55),*  *Providencia stuartii (ATCC29916, NAE16)* | *Entada abyssinica* Steud. ex A. Rich. (L, R),  *Entada Africana*  Guill. & Perr. (B),  *Carapa procera* DC. (B),  *Carica papaya* L. (seed),  *Persea americana* Mill. (Stones) | Fabaceae,  Fabaceae,  Méliaceae  Caricaceae  Lauraceae | CH2Cl2/MeOH (1:1) ethyl acetate, Methanol | colour changes, MIC & MBC) |
| 4 | Romha *et al*, 2017 | AWD and AD | *S. aureus* 25923,  *P. aeruginosa 27853,* and  *E. coli 20922* | *Calpurnia aurea* (Aiton) Benth. (Air.)*,*  *Croton macrostachyus* Hochst. ex Delile (L),  *Withania somnifera* (L.) Dunal (L.), | Fabaceae,  Fabaceae, Euphorbiacea, Solanaceae,  Fabaceae, &  Solanaceae | Methanol (99.8%) and chloroform (95%) | ZI & Bacterial growth (MIC & MBC) |

| 5 | Taye *et al*, 20111 | AWD & MiD | *S. aureus, (ATCC 25923),*  *S. pyogens, (ATCC 19615)*  *E. coli, (ATCC 25922),*  *P. aeruginosa, (27853),*  *Proteus vulgaris and (PROVU-01).* | *Achyranthes aspera* L.(L),  *Brucea antidysenterica* J.F.Mill. (R),  *Datura stramonium* L.(L),  *Croton macrostachyus* Hochst. ex Delile (L),  *Acokanthera schimperi* (A.DC.) Schweinf. (L),  *Phytolacca dodecandra* L'Hér. (R),  *Millettia ferruginea* (Hochst.) Hochst. ex Baker (L), and  *Solanum incanum* L. (L). | Amaranthaceae,  Simaroubaceae,  Solanaceae, Euphorbiaceae,  Apocynaceae,  Phytolaccaceae,  Fabaceae &  Solanaceae | Methanol & aqueous | ZI and bacterial growth (MIC & MBC) |  |
| --- | --- | --- | --- | --- | --- | --- | --- | --- |
| 6 | Belal *et al,* 2017 | Agar DD | E*. coli,*  *Salmonella Typhi,*  *Proteus vulgaris,*  *Klebsiella pneumonae,*  *Enterococcus faecalis and Staphylococcus aureus.* | *Cumin cyminum* L. (S) | *Apiaceae* | Essential oil | ZI |  |
| 7 | Baynesagne *et al*, 2017 | MAD | *E. coli* *(ATCC 25922),*  *S. aureus* *(ATCC 25923),*  *Streptococcus pneumoniae* *(ATCC 63),*  *E. coli (isolate),*  *Klebsiella pneumoniae* (isolate) and  *S. pneumoniae* (isolate) | *Datura stramonium* L. (L) | *Solanaceae* | Ethanol, methanol, acetone, chloroform and distilled water | Growth of microorganisms (MIC & MBC) |  |
| 8 | Vazirian *et al*, 2016 | MiD & DD | *S. aureus ATCC 29213,*  *E. coli ATCC 25922,*  *Pseudomonas aeruginosa ATCC 27853*, *Enterococcus faecalis ATCC 29212,*  *Salmonella typhimurium* (Isolate) and  *E. coli* (Isolate) | *Trachyspermum ammi* (L.) Sprague (S) | *Apiaceae* | Essential oil | ZI & bacterial growth (MIC & MBC) |  |
| 9 | Yeabyo *et al*, 2018 | AWD Assay | *E. coli (ATCC 25922),*  *Enterobacter aerogenes (ATCC 13048),*  *Klebsiella pneumoniae, (ATCC 700603),*  *Vibrio cholera. (ATCC 39315),*  *Bacillus subtilis (ATCC 3915),*  *Enterococcus faecalis (ATCC 29212)* | *Verbascum sinaiticum*  Benth. (R) | *Scrophulariaceae* | Diethyl ether,  chloroform, acetone, and  ethanol | ZI |  |
| 10 | Begashawu *et al*, 2016 | AWD & BMD assay | *S. aureus DSM 7246, Salmonella typhimurium ATCC 13311, Aeruginosa DSM 1117 and E. coli ATCC 25722* | *Kosteletzkya begonifolia* (Ulbr.) Ulbr (L)  *Leucas martinicensis (Jacq.) R.Br.*(L) and  *Ranunculus multifidus*Forssk. (L) | *Ranunculaceae, Lamiaceae & Malvaceae respectively* | Petroleum ether, Chloroform, methanol for all the three plants | ZI and Bacterial growth (MIC & MBC)) |  |
| 11 | Burt *et al,* 2003 | DD assay, MiD (Colorimetric) | *E. coli O157:H7* | *Pimenta racemose* (Mill.) J.W.Moore (L*), Eugenia caryophyllata*,Thunberg (L)  *Origanum vulgare* L. (L) and  *Thymus vulgaris* L. (L) | *Myrtaceae, Myrtaceae, Lamiaceae & Respectively* | Essential oil | ZI & bacterial growth (MIC & MBC) |  |
| 12 | Chaieb *et al,* 2011 | MID & Crystal Violet assay | *E. coli ATCC 35218,*  *Salmonella enterica serovar Typhimurium ATCC 14028*,  *P. aeruginosa ATCC 27853*,  Vibrio *alginolyticus ATCC 33787,*  *Vibrio parahaemolyticus ATCC 17802,*  *Bacillus cereus ATCC 14579,*  *Listeria monocytogene ATCC 19115,*  *Enterococcus faecalis ATCC 29212,*  *Micrococcus luteus NCIMB 8166,*  *S. aureus ATCC 25923* &  *S. epidermidis CIP 106510* | Thymoquinone (compound) obtained from *Nigella sativa* L. (S) | *Ranunculaceae* | Compound | Bacterial growth & Colour changes (MIC & MBC) |  |
| 13 | Bisht *et al,* 2014 | AWD test | *S. aureus* (MRSA and MSSA),  *Enterococcus spp.* and  *Streptococcus spp* | *Withania somnifera* (L.) Dunal (L) | *Solanaceae* | Methanol | ZI |  |
| 14 | Djeussi *et al,* 2016 | MiD (colorimetric) | *E. coli (ATCC8739, ATCC10536, AG100, AG100A, AG100ATet, AG102, MC4100 W3110),*  *Enterobacter aerogenes (ATCC13048, CM64, EA27, EA289, EA298, EA294),*  *Klebsiella pneumoniae (ATCC11296, KP55, KP63, K24, K2),*  *Enterobacter cloacae (ECCI69, BM47, BM67),*  *P. aeruginosa (PA01, PA124) and*  *Providencia stuartii (ATCC29916, NEA16, PS2636, PS299645)* | *Anthocleista schweinfurthii* Gilg (B, F &L),  *Nauclea latifolia* Sm (B, F & L),  *Boehmeria platyphylla* Buch. -Ham. ex D. Don (W),  *Caucalis melanantha* (Steud. ex Hochst.) Benth. & Hook. ex Hiern (W),  *Erigeron floribundus* (Kunth) Sch.Bip. (W) and  *Zehneria scobra* (L.f.) Sond*.* (W*)* | *Loganiaceae,* Gentianaceae, Apiaceae | Maceration | Bacterial growth (colour changes, MIC and MBC) |  |
| 15 | Asres *et al*, 2006 | MAD & DD | *E. coli (K99, K88, 306, LT37, 872, ROW 7/12, 3:37C, CD/99/1),*  *Salmonella typhi Ty2,*  *Shigella dysentery 1, Dysentery 8, S. soneii 1, S. boydii D13629 and S. flexneri Type 6,*  *Vibrio cholerae 1313, 293, 1315 and 85,*  *S. aureus ML 267,*  *Bacillus pumilus 82* and  *B. subtilis ATCC 6633* | *Combretum molle* R.Br. ex G.Don (B) | *Combretaceae* | Petroleum ether, dichloromethane, acetone and methanol | MIC & MBC and ZI |  |
| 16 | Sileshi *et al,* 2008 | AD & DD | *S. aureus (Isolate & ATCC 25923), Streptococcus pyogenes (isolate),*  *E. coli (ATCC 2590),*  *P. aeruginosa (isolate & ATCC 27853)* | *Clerodendrum myricoides* (Hochst.) Steane & Mabb. (L),  *Ficus plamata* Forssk. (L),  *Grewia ferruginea* Hochst. ex A. Rich. (L)  *Periploca linerifolia* Quart. -Dill. & A.Rich. (aerial) | *Lamiaceae, Moraceae, Tiliaceae, Asclepediaceae* | 80% Methanol extract, petroleum ether, chloroform, acetone and methanol fractions | ZI |  |
| 17 | Habtamu *et a*l, 2018 | Agar DD | *E. coli,*  *K. pneumoniae,*  *Proteus mirabili,*  *S. aureus* and *bacillus* | *Vernonia amygdalina* (Delile) Sch.Bip. (F) | *Asteraceae* | Hexane, chloroform, and acetone | ZI |  |
| 18 | Adedapo et *al,* 2008 | MD & DD | *Bacillus cereus,*  *S. epidermidis,*  *S. aureus,*  *Micrococcus kristinae, and*  *Streptococcus pyogens,*  *E. coli,*  *Salmonella pooni,*  *Serratia marcescens,*  *P. aeruginosa*, and  *K. pneumoniae* | *Calpurnia aurea* (Aiton) Benth. (S, L) | *Legumes* | methanol | ZI & bacterial growth (MIC & MBC) |  |
| 19 | Habtamu et *al,* 2017 | AD & Agar DD | *S. aureus,*  *E. coli,*  *P. aeruginosa,*  *S. boydii* and  *S. typhi* | *Clematis hirsuta* Guill. & Perr. (L) | *Ranunculaceae* | 80% methanol and chloroform | ZI and (MIC &MBC) |  |
| 20 | Dua *et al*, 2013 | AD and Agar DD | *E. coli (MTCC96),*  *P. aeruginosa (MTCC741),*  *S. aureus (MTCC96),*  *Bacillus Pumilus (MTCC7411),* | *Cuminum Cyminum* L. (S) | *Umbellifers* | 80% methanol | ZI and (MIC & MBC) |  |
| 21 | Umer *et al*, 2013 | AWD | *Salmonella spp. (Typhi, Paratyphi, Typhimurium)*  *Shigella species,*  *P. aeruginosa,*  *S. aureus* and  *E. coli* | *Calpurnea aurea* (Aiton) Benth. (L) | *Fabaceae* | 80% methanol | ZI |  |
| 22 | Vijayasanthi *et al*, 2014 | DD & MAD | *Bacillus cereus MTCC 442,*  *E. coli MTCC 598,*  *K. pneumoniae MTCC 7407,*  *P. aeruginosa MTCC 42642*  *Proteus vulgaris MTCC 742*  *S. aureus MTCC 3160,*  *Salmonella typhi MTCC 3917,*  *Shigella flexneri MTCC 1457*  *Streptococcus pneumonia MTCC 655,* | *Delonix elata* (L.) Gamble (L)  *Spathodea campanulata* P.Beauv. (L) | *Fabaceae*  *Bignoniaceae* | Aqueous, Methanol extracts | ZI and (MIC) |  |
| 23 | Tadeg *et al*, 2005 | AWD & MiD | *S. aureus (ATCC 6538),*  *E. coli (ATCC 25922)* &  *P. aeruginosa (ATCC 27853)* | *Acokanthera schimperi* (A.DC.) Schweinf. (L),  *Calpurnia aurea* (Aiton) Benth. (L),  *Kalanchoe petitiana* A. Rich. (L),  *Lippia adoensis* Hochst (L),  *Olinia rochetiana* A.Juss (L),  *Verbascum erianthum Benth*  (L),  *Phytolacca dodecandra* L’Hér. (F) &  *Malva parviflora* L. (R) | *Apocynaceae, Leguminosae, Crassulaceae, Verbenaceae, Malvacea,*  *Oliniacea, Phytolaccaceae, Scrophulariaceae* | 80% methanol extract; petroleum ether, chloroform, acetone and methanol fractions | ZI and (MIC & MBC) |  |
| 24 | Mwitari *et a*l, 2013 | DD & AD | *S. aureus ATCC 25923*,  clinical isolate MRSA,  *E. coli ATCC 25922* and  *P. aeruginosa ATCC 27853*. | *Withania somnifera* (L.) Dunal (Ar),  *Prunus Africana* (Hook.f.) Kalkman *(SB)*,  *Warbugia ugandensis* Sprague (SB) and  *Plectranthus barbatus* Andrews (SB) | Solanaceae | Dichloromethane, ethyl acetate, methanol | ZI, MIC & MBC |  |
| 25 | Awino *et a*l, 2007 | DD | *Salmonella species,*  *Proteus spp.,*  *P. aeruginosa,*  *K. pneumoniae,*  *E. coli,*  *Cryptococcus neoformans,*  *Shigella dysentiae* and  S. aureus | *Embelia schimperi* Vatke (SB) | *Myrsinaceae* | Ethyl acetate a compound | ZI, (MIC) |  |
| 26 | Seshathri *et al,* 2011 | AWD | *Streptococcus pyogenes (ATCC 19615)* | *Clausenia anisate* (Willd.) Hook.f. ex Benth. (S), *Clematis simensis* Fresen. (S), *Cleodendrum myricoides* (Hochst.) Steane & Mabb. (S),  *Juniperus procera* Hochst. ex Endl. (S),  *Justicia schimperiana* (Hochst. ex Nees) T. Anderson (S), *Olea europea* L. (S),  *Phoenix reclinata* Jacq. (Petiole), Rubus *apitalus* Poir. (S),  *Sesbania sesban* (L.) Merr. (S), *Sida rhombifolia* L. (S), *Spilanthes mauritiana* Delile (F), *Stereospermum kunthianum* cham.(S)  *Vernonia amygdalina* (Delile) Sch.Bip*.* (L) | *Rutaceae, Ranunculaceae, Verbanaceae, Cuprassaceae, Acanthaceae,*  *Oleacea,*  *Arecaceae,*  *Rosaceae,*  *Fabaceae,*  *Malvaceae, Compositae, Bignoniaceae* & *Asteraceae* | Ethanol, aqueous | ZI |  |
| 27 | Obey *et al,* 2016 | AWD | *E. coli ATCC 25922*,  *Enterobacter*,  *K. pneumoniae ATCC 1583380*,  *P. aerogenes MTCC 2990*  *Salmonella typhi ATCC 2202*, | *Croton macrostachyus* Hochst. ex Delile (SB) | *Euphorbiaceae* | Methanol, ethyl acetate, n-butanol | ZI |  |
| 28 | Ewansiha *et al,* 2012 | DD & MaD | *S. aureus,*  *E. coli,*  *Salmonella typh*i | *Cymhopogon Citratus* (DC.) Stapf (L) | *Poaceae* | Hexane, Chloroform and Methanol | ZI, MIC |  |
| 29 | Ameya *et a*l, 2016 | DD assay and AD | *E. coli (ATCC-25922)*  *E. faecalis (ATCC-29212)*  S*. aureus (ATCC-25923),* | *Echinops kebericho* Mesfin (R) | *Asteraceae* | Ethanol. methanol. aqueous | ZI, MIC |  |
| 30 | Singh *et al,* 2011 | AD and DD | A total of 1093 bacterial strains of 26 genera | *Cymhopogon Citratus* (DC.) Stapf (L) | *Poaceae* | essential oil | ZI, MIC |  |
| 31 | Duraipandiyan *et al,* 2012 | MiD and DD assay | *Bacillus subtilis MTCC 441,*  *E. coli ATCC 25922,*  *Enterococcus faecalis ATCC 29212,*  *Erwinia sp. MTCC 2760*  *K. pneumoniae ATCC 15380,*  *P. aeruginosa ATCC 27853* and  *Proteus vulgaris MTCC 1771,*  *S. aureus ATCC 25923,*  *S. epidermidis MTCC 3615,* | *Costus speciosus* (J.Koenig) S.R.Dutta (Rh) | *Costaceae* | Hexane, chloroform, ethyl acetate, methanol and water | ZI, MIC |  |
| 32 | Ameya *et al*, 2015 | DD, AD | *S. aureus (ATCC-25923),*  *E. faecalis (ATCC-29212)* and  *E. coli (ATCC-25922)* | *Taverniera abyssinica* A.Rich*.* (R) | *Fabaceae* | Ethanol, methanol and distilled water | ZI, MIC |  |
| 33 | Hassanshahian *et al,* 2014 | Agar DD and MiD | *S. aureus,*  *E. coli*  *K. pneumoniae* | *Trachyspermum ammi* (L.) Sprague (S) | *Umbellifers* | Essential oil | ZI, MIC |  |
| 34 | Lulekal *et al,* 2014 | MiD (MIC) | *Bacillus cereus ATCC 11778,*  *Bacteroides fragilis ATCC 25285,*  *Candida albicans ATCC 10231, Clostridium perfringens DSM 11778,*  *E. coli ATCC 25922,*  *E. faecalis ATCC 29212,*  *L. monocytogenes ATCC 7644,*  *P. aeruginosa ATCC 27853,*  *S. aureus ATCC 29213,*  *S. epidermidis ATCC 12228,*  *S. enteritidis ATCC 13076,* and  *Streptococcus pyogenes ATCC 19615* | *Bersama abyssinica* Fresen. *(*L),  *Calpurnia* aurea (Lam.) Benth (R),  *Carissa spinarum* L. (R),  *Clematis hirsuta* Guill. & Perr. (L),  *Clutia abyssinica* Jaub. & Spach (R),  *Croton* macrostachyus Hochst. ex Delile (L),  *Cyathula cylindrica* Moq. (R),  *Dodonaea angustifolia* (L.f.) J.G.West (L),  *Embelia schimperi* Vatek (S),  *Jasminum abyssinicum* Hochst. ex DC. (L),  *Maesa lanceolata* Forssk (L),  *Ocimum lamiifolium* Hochst. ex Benth. (L),  *Olinia rochetiana* A.Juss. (L),  *Rubus steudneri* Schwein (R),  *Rumex nepalensis* Spreng (R),  *Thalictrum rhynchocarpum* Quart-Dill. & A.Rich. (R),  *Verbascum sinaiticum* Benth. (L),  *Vernonia amygdalina* (Delile) Sch.Bip. (F) | *Melianthaceae, Fabaceae,*  *Apocynaceae, Ranunculaceae, Euphorbiaceae, Amaranthaceae, Sapindaceae, Myrsinaceae, Oleaceae,*  *Lamiaceae, Oliniaceae,*  *Rosaceae, Polygonaceae, Ranunculaceae, Scrophulariaceae, Asteraceae* | Methanol | ZI, MIC |  |
| 35 | Bacha *et al*, 2016 | MiD & AWD | *E. coli K12, DSM 498,*  *P. aeruginosa DSM 1117,*  *S. aureus DSM 346,*  *Bacillus cereus ATCC 10987,*  *B. cereus* (isolate*)* | *Aframomum corrorima* (A.Braun) P.C.M.Jansen (F),  *Albiza schimperiana* Oliv. (R),  *Curcuma longa* L. (Rh),  *Erythrinia brucei* Schweinf. emend. Gillett (SB),  *Justica schimperiana*  (Hochst. ex Nees) T. Anderson (S),  *Nigella sativa* L. (S),  *Ocimum sauve* Wild. (L) &  *Vernonia amygdalina* (Delile) Sch.Bip. (L), | *Zingiberaceae, Leguminosae, Zingiberaceae, Leguminosae, Acanthaceae,*  *Ranunculaceae* *Lamiaceae* | Petroleum ether, chloroform, methanol,  water and oilo resins | ZI, MIC |  |
| 36 | Njeru *et al*, 2015 | Agar DD & MiD | *S. aureus (ATTC 25923*),  MRSA strain (isolate);  *E. coli (ATTC 25922)*,  *K. pneumoniae* (isolate),  *P. aeruginosa (ATCC 27853)*, *Salmonella typhi* (isolate),  *Shigella sonnei* (isolate) | *Premna resinosa* (Hochst.) Schauer (R) | *Verbenacea*e | Aqueous, methanol; petroleum ether. Then Petroleum ether was fractionated to --ethyl acetate, chloroform, methanol | ZI, MIC |  |
| 37 | Ngeny  *et al,* 2013 | Agar diffusion & MiD | *P. aeruginosa ATCC 27853,*  *S. aureus ATCC 25923,*  *E. coli ATCC 25922,*  *K. pneumoniae* (isolate),  *MRSA* (isolate). | *Hagenia abyssinica* (Bruce) J.F.Gmel. (L & SB),  *Fuerstia africana* T.C.E.Fr. (Ae),  *Ekebergia capensis* Sparrm (R)  *Asparagus racemosus* Wild. (SB) | *Rosaceae,*  *Lamiaceae, Asparagaceae Meliaceae* | Hexane, dichloromethane, methanol, aqueous | ZI, MIC |  |
| 38 | Hussien *et al,* 2011 | AWD method | *S. aureus (ATCC 25923),*  *E. coli (ATCC 25922),*  *S. typhi (ATCC 83859),*  *S. aeruginosa (ATCC 27853)* | *Brassica Nigra* L. (S),  *Thymus shimperi* Ronniger  (L),  *Ocimum basilicum* L. (L),  *Syzygum aromaticum* (L.) Merr. & L.M.Perry (F),  *Electtaria Caradamom* (L.) Maton  (F) &  *Cinnamon Zeylanicum* J.Presl (SB) | *Brassicaceae, Lamiaceae, Lamiaceae, Myrtaceae, Zingiberaceae & Lauraceae* respectively | Hydrosol prepared from plurized plant parts | ZI, MIC |  |
| 39 | Debalke *et al*, 2018 | AWD & MiD | E*. coli (ATCC-27853),*  *S. typhi (ATCC -13062),*  *S. aureus (ATCC- 2529),*  *K. pneumonia* and *Citrobacte*r. | *Sida rhombifolia* L. (W) | *Malvaceae* | 80% Methanol | ZI, MIC |  |
| 40 | Unnithan *et al*, 2013 | AWD | *E. coli* (isolate) and  *S. aureus* (isolates) | *Ocimum basilicum* L. (Ae) | Lamiaceae | Essential oil | ZI |  |
| 41 | Meshesha *et al,* 2017 | AWD | *S. aureus (ATCC 25923),*  *Enterococcus faecalis (ATCC 29212),*  *E. coli (ATCC 25922)* and  *P. aeruginosa (ATCC 27853)* | *Kniphofia Moench* (L.) Oken (L) | *Asphodelacea* | Chloroform/methanol (1:1),  Ethyl acetate/water in (9:1),  Ethyl acetate;  Compounds: Chrysophanol, 3,5,8-trihydroxy-2methylnaphthalen-1,4-dione, asphodeline, 10-hydroxy-10,7ꞌ(chrysophanolanthrone) chrysophanol | ZI |  |
| 42 | Gadisa *et al,* 2019 | MiD | *E. coli (ATCC25922),*  *K. pneumoniae (ATCC700603)*  *S. aureus (ATCC25923),* | *Blepharis cuspidata* Lindau  (L), *Boswellia ogadensis* Vollesen (L) and *Thymus schimper* Ronniger (L) | *Acanthaceae, Burseraceae* and *Lamiaceae* | Essential oil | MIC and MBC |  |
| 43 | Belay *et al*, 2011 | MaD | *Bacillus cereus,*  *Citrobacter spp,*  *E. coli (ATCC 25922),*  *K. pneumonia* and  *Listeria monocytogenes,*  *P. aeruginosa (ATCC 27853),*  *Proteus mirabilis.*  *S. aureus (ATCC 25923),*  *Salmonella paratyphi,*  *Shigella dysenteriae,*  *Streptococcus pyogenes,* | *Artemisia abyssinthium* L. absinthium (L), Artemisia abyssinica (L),  *Croton macrostachyus* Hochst. ex Delile (L), *Echnops kebericho* Mesfin (Tu) and *Satureja puncatat* (Benth.) R.Br. ex Briq.  (Ber) | *Asteraceae, Euphorbiaceae, Asteraceae & Lamiaceae* respectively | Essential oil | MIC & MBC |  |
| 44 | Mulat *et al,* 2015 | AWD & MaD | *E. coli (ATCC25722), S. aureus (ATCC25903), Shigella sonei (ATCC259131)* and *Salmonella typhimurium (ATCC13311)* | *Ocimum sauve* Willd (L), *Ruta graveolens* L. (L), *Ocimum lamiifolium* Hochst. ex Benth. (L), *Nigella sativa* L. (S) &  (L). | *Lamiaceae, Rutaceae, Lamiaceae, Ranunculaceae* & *Asteraceae* | Petroleum ether,  Chloroform and  Methanol, essential oil | ZI, MIC & MBC |  |
| 45 | Chalo *et al,* 2015 | AWD, MD & DD | P*. aeruginosa, E. coli, methicillin resistant S. aureus (MRSA), Bacillus cereus* | *Schrebera alata* (Hochst.) Welw. (B), *Ormocarpum kirkii* S.Moore (Ae), *Cussonia holstii* Harms ex Engl. (B) & *Helichrysum forskahlii* (J.F.Gmel.) Hilliard & B.L.Burtt (W) | *Oleaceae, Fabaceae, Araliaceae & Asteraceae* respectively | Dichloromethane,  Methanol,  Aqueous | ZI, MIC |  |
| 46 | Habtamu *et al,* 2017 | AD & DD assay | *S. aureus, Salmonella typhi, E. coli, P. aeruginosa* and *Shigella boydii* | *Achyranthes aspera* L. (L) | *Amaranthaceae* | Chloroform,  Methanol | ZI, MIC |  |
| 47 | Genanew *et al,* 2017 | AWD & MaD | *S. aureus (ATCC 25923), Streptococcus pneumonia (ATCC 49619), and Streptococcus pyogenes (ATCC 19615), E. coli (ATCC 25922), P. aeruginosa (ATCC 2706), K. pneumonia (ATCC 700603)* and *Salmonella typhi (ATCC 1912/R)* | *Aloe macrocarpa* Tod (L). | *Aloaceae* | Latex and gel | ZI, MIC |  |
| 48 | Hagos *et al,* 2017 | AWD | *E. coli (MTCC 40),*  *P. aeruginosa (MTCC 424),*  *P. vulgaris (MTCC 742),*  *S. aureus (MTCC 87)*  *S. faecalis (MTCC5383)* | *Moringa stenopetala* (Baker f.) Cufod. (L) | *Moringaceae* | Methanol and Aqueous | ZI |  |
| 49 | Ameya *et al*, 2018 | MaD &, AWD | *E. coli, Klebsiella species* and *S. aureus* | *Nicotiana tabacum* L. (L) | *Euphorbiaceae* | Petroleum ether, chloroform, diethyl ether, ethyl acetate, acetone, dichloromethane and methanol | ZI, Mic and MBC |  |
| 50 | Abew *et al,* 2014 | AWD & MaD | *E. coli (Isolate and ATCC 25922)*  *S. aureus (MRSA and ATCC 2923),* | *Zehneria scabra* (L.f.) Sond. (L) & *Ricinus communis* L. (L) | *Curbitaceae, Euphorbiaceae* | Benzen (1), chloroform/ acetone with ratio 1:1 (2); 70% alcohol (3) and distilled water (4) | ZI, MIC and MBC |  |
| 51 | Mummed *et al,* 2018 | AWD & MaD | *S. aureus ATCC) 25923, P. aeruginosa ATCC 27853, E. coli ATCC 25922* and *K. pneumoniae ATCC 700603* | *Cissus quadrangularis* L. (Ae), *Commelina benghalensis* L. (L), *Euphorbia heterophylla* L.(R), *Euphorbia prostrate* Aiton (W), *Grewia villosa* Willd. (L), *Momordica schimperiana* Naudin (F), *Trianthema*  *portulacastrum*L. (ae), *Schinus molle* L. (L), and *Solanum incanum* L. (F). | *Vitaceae, Commelinaceae, Euphorbiaceae, Euphorbiaceae, Malvaceae, Cucurbitaceae, Aizoaceae, Anacardiaceae* & *Solanaceae* respectively | Methanol extract | ZI, MIC & MBC |  |
| 52 | Minale *et al,* 2014 | AWD & MaD | *Bacillus pumillus 82,*  *B. subtilis ATCC 6633 and*  *S. aureus ML 267,*  *E. coli (K99, K88, CD/99/1, LT37, 306, 872, ROW 7/12, 3:37C),*  *Salmonella enterica TD 01, S. typhi Ty2,*  *Shigella boydii D13629, S. dysentery 8, S. flexneri Type 6, S. soneii 1,*  *Vibrio cholerae (85, 293, 1313* and *1315)* | *Aloe sinana* Reynolds (L) | *Asphodelaceae* | Leave latex … compound (anthrones) | ZI, MIC & MBC |  |
| 53 | Moglad *et al*, 2014 | AWD & MaD | *Bacillus subtilus (NCTC 8236),*  *E. coli ATCC 25922*,  S*. aureus (ATCC 25923),*  *Salmonella typhi NCTC 0650)* | *Maerua oblongifolia* (Forssk.) A.Rich*.* (L & St) | *Capparaceae* | Chloroform, Methanol, | ZI, MIC & MBC |  |
| 54 | Hawaze *et al*, 2012 | Paper DD & MaD | *S. aureus* ATCC 25923,  *P. aeruginosa* ATCC 27853 | *Clematis longicauda* Steud. ex A.Rich.  (L) *Clematis burgensis* Guill. & Perr. (L) | *Ranunculaceae* | Petroleum ether, Methanol | ZI, MIC & MBC |  |
| 55 | Regassa *et al,* 2012 | DD & MaD | *S. aureus* and  *S. agalactiae* | *Combretum molle* R.Br. ex G.Don (L, B, St, S) | *Combretaceae* | 90% Ethanol | ZI, MIC & MBC |  |
| 56 | Zulfa *et al,* 2015 | MiD & Paper DD assay | *Bacillus cereus ATCC 10987, E. coli O157:H7 ATCC 25922, K. pneumoniae ATCC 15692, Candida albicans ATCC 10231* | *Cymbopogon citratus* (DC.) Stapf (L) | *Poaceae* | 100% (v/v) Methanol | ZI, MIC & MBC |  |
| 57 | Ameya *et al,* 2018 | AWD & BMiD | *S. aureus* (MRSA and ATCC® 25923™),  *Salmonella enterica subsp. enterica (ATCC® 13311™) and*  *K. pneumoniae (ATCC® 700603™),*  *P. aeruginosa* and  *E. coli* | *Capsicum frutescens* L. (F) | *Solanaceae* | Distilled water, Acetone, Ethanol, Methanol, Ethyl acetate, and Chloroform | ZI, MIC & MBC |  |
| 58 | Teka *et al*, 2015 | BMiD | *E. coli ATCC 25922,*  *E. faecalis ATCC 29212,*  *P. aeruginosa ATCC 27853,*  *S. aureus (ATCC 25923, ATCC 29213, ATCC 33591, ATCC 33592, ATCC 43300, ATCC BAA 976), and*  *S. epidermidis ATCC 12228.*  clinical isolates of antibiotic-sensitive *S. aureus* strains *(SA1, SA2, SA3, SA4, SA5, SA6, SA7, SA8, SA9, SA10)* | *Apodytes dimidiata* E.Mey. ex Arn. (SB); *Asparagus africanus* Lam. (L), *Bersama abyssinica* Fresen (S), *Cucumis ficifolius* A.Rich. *(*R), *Gladiolous abyssinicus*(Brongn. ex Lem.) N.E.Br.  (Bu) *Guizotia schimperi* Sch.Bip. (L*),*  *Lippia adoensis* Hochst (L),  *Olinia rochetiana* A. Juss. (SB), *Pavonia urens* Cav.  (L),  *Premna schimperi* Engl. (L),  *Pittosporum viridiflorum* Sims. (L),  *Polygala sadebeckiana* Hoffmanns. & Link (R), *Sida rhombifolia* L.(R),  *Solanum incanum* L. (F) | *Icacinaceae, Asparagaceae, Melianthaceae, Cucurbitaceae, Iridaceae, Asteraceae, Verbenaceae, Oliniacea, Malvacea, Lamiaceae, Pittosporaceae, Polygalaceae, Malvaceae & Solanaceae* respectively | 80 % Ethanol | MIC & MBC |  |
| 59 | Asamenew *et al,* 2011 | DD & MiD | *Bacillus subtilis (ATCC 6633),*  *B. pumillus,*  *E. coli (K99, K88, CD/99/1, LT37, 306, 872, ROW 7/12, 3:37C),*  *S. aureus (ML 267, NCTC 11994),*  *Salmonella typhi (Ty2),*  *S. typhimurium (ATCC 1255),*  *Shigella boydii (D3629), S. flexneri, (Type 6), S. soneii (1), S. dysentery (1), S. dysentery,*  *Vibrio cholerae (85, 293, (1313, 1315).* | *Aloe harlana* Reynolds (L) | *Asphodelacea*e | Latexa 7‐OMA & Aloin | ZI, MIC& MBC |  |
| 60 | Kalayou *et al,* 2012 | DD & MiD | *E coli,*  *K. pneumonae*  *S. aureus, S. intermedius, S. hicus,* | *Achyranthes aspera* L.(L), *Ficus caria* L. (L*),*  *Malvi parviflora* L. (L), *Vernonia species* (Willd.) Drake (L),  *Solanum hastifolium* Hochst. ex Dunal (L), *Calpurinia aurea* (Aiton) Benth. (L), *Nicotiana tabacum* L. (L), *Ziziphus spina-christi* (L.) Desf. (L, S), *Croton macrostachys* Hochst. ex Delile (L) | *Asparagaceae, Moraceae, Malvaceae, Asteraceae, Solanaceae, Fabaceae, Solanaceae, Rhamnaceae* & *Euphorbiaceae* respectively | 70% Methanol | ZI, MIC & MBC |  |
| 61 | Begashaw *et al,* 2017 | AWD & MiD | *E. coli (ATCC1925525),*  *K. pneumoniae (ATCC70060),*  *P. aeruginosa (ATCC27853),*  P. *mirabilis (ATCC12386)*  *S. aureus (ATCC2923),*  *S. pneumoniae (ATCC137348)*,  *S. pyogenes (ATCC19615),* | *Hibiscus micranthus* L. (L) | *Malvaceae* | 80% Methanol | ZI, MIC & MBC |  |
| 62 | Bekele *et al*, 2015 | AWD & MiD | *E. coli (ATCC 25922),*  *K. pneumoniae (isolate*),  *S. aureus (MRSA* and *ATCC 25923*),  *Shigella flexneri* *(ATCC 12022*),  *S. pneumoniae (ATCC 63* and isolate) | *Thymus schimperi* Ronniger  (L) | *Lamiaceae* | Chloroform, ethanol, methanol, and aqueous | ZI & MIC & MBC |  |
| 63 | Palla *et al*, 2015 | AWD | Bacillus cereus,  *E. coli 018: K1:H7, strain RS218*,  *Enterococcus faecalis* (VRE), and  *P. aeruginosa*.  *S. aureus (MRSA),*  *Salmonella typhi (ATCC 14028*) | *Linum usitatissimum* L. (S) | *Linaceae* | 70% Methanol | ZI |  |
| 64 | Albejo *et al,* 2015 | MiD | *S. aureus* | *Vernonia auriculifera* (Hiern) Isawumi (L) | *Compositae* | N-hexane, chloroform, methanol, and water | MIC & MBC |  |
| 65 | Abdissa *et al*, 2015 | Paper DD | *E. coli,*  *Bacillus subtills*  *Salmonella typhi,*  *S. aureus* | *Aloe pulcherrima* M.G.Gilbert & Sebsebe (L) | *Asphodelaceae* | N-hexane, chloroform, acetone and methanol; three compounds) | ZI |  |
| 66 | Girmay *et a*, 2017 | DD | *B. subtilis (ATCC 6633)*  *E. coli (ATCC 35218),*  *P. aeruginosa (ATCC 27853)* and  *S. aureus (ATCC 25923),* | Lepidium Sativium L. (S) | *Cruciferae* | N-hexane, chloroform/ methanol (1:1 | ZI |  |
| 67 | Seid*,* 2016 | Paper DD | *E. coli*  *S. aureus*  *Shigella flexneri*  *Streptococcus pyrogenes,* | *Foeniculum vulgare* Mill. (L) | *Umbelliferae* | Petroleum ether, Chloroform, Chloroform-methanol (1:1), Methanol | ZI |  |
| 68 | Teshome *et al,* 2018 | AWD | *E. coli ATC25922,*  *P. aeruginosa ATCC27853*  *S. aureus ATCC 25923,*  *S. typhi NTCC83859,* | *Clematis simensis* Fresen (L) | ‎*Ranunculaceae* | Petroleum ether (60-80°C), acetone and methanol | ZI |  |
| 69 | Girmay *et al,* 2015 | Paper DD | *Bacillus subtilis,*  *E. coli* and  *Salmonella typhi*  *S. aureus* | *Datura stramonium* L. (L) | *Euphorbiaceae* | Chloroform, ethanol, hexane, petroleum ether, and acetone | ZI |  |
| 70 | Nyanchoka*,* 2016 | Paper DD | *Bacillus subtilis*  *diarrheagenic E. coli*  *K. pneumoniae,*  *Penicillium notatum,*  *P. aeruginosa*  *Salmonella typhi,*  *Shigella dysentriae,*  *Vibrio cholorae,* | *Bersama abyssinica* Fresen. (SB) | *Melanthiaceae* | N-hexane, Dichloromethane, ethyl acetate, methanol | ZI |  |
| 71 | Goji *et al,* 2006 | Hole-plate assay | *E. coli (ATCC 25922),*  *P aeruginosa (Isolate, ATCC 27853),*  *S. aureus (Isolate, ATCC 25923), Streptococcus pyogenes* (isolate) | *Jasminum abyssinicum* Hochst. ex DC. (L), *Solanecio gigas* (Vatke) C. Jeffrey (L)  Lagenaria *siceraria* (Molina) Standl. (L, S &F) | *Oleaceae, Asteraceae, Cucurbitaceae* |  | ZI |  |
| 72 | Megeressa *et al*, 2015 | DD & MiD | *Bacillus pumilus 82*,  *B. subtilis ATCC 6633,*  *S. aureus ML 267*,  *E. coli (3:37C*, *7360*, *872*, *CD/99/1*, *K 88*, *T37*, *ROW* *7/12*, *5933)*,  *Salmonella enterica TD 01*, *S. typhi Ty2*, *Shigella boydii D13629*, *S. dysentery 8, S. flexneri Type 6, S. soneii 1,*  *Vibrio cholerae (NCTC 5596, NCTC 10732, NCTC 11501, & NCTC 4693* | *Aloe trigonantha* L.C.Leach  (L) | *Aloaceae* | Aloesin (1),  8-O-Methyl-7-hydroxyaloin A/B (2),  Aloin A/B (3);  Aloin-6’-O-acetate A/B (4); | ZI and MIC |  |
| 73 | Hussien *et al,* 2010 | AWD | *S. aureus ATCC 25923*,  *E. coli ATCC 25922*,  *P. aeruginosa ATCC 27853* | *Pycnostachys abyssinica* Fresen (L, S, R)  *Pycnostachys eminii* Gürke (L, S, R) | *Laminaceae* | Essential oils;  petroleum ether chloroform and methanol | ZI |  |
| 74 | Delelegn *et al,* 2018 | AWD & AD | *E. coli (ATCC 2592*, isolate),  *Salmonella typhi* (isolate) and  *Shigella dysenteriae* (isolate) | *Moringa oleifera* Lam. (S) | *Moringaceae* | Methanol, acetone and aqueous | ZI, MIC & MBC |  |
| 75 | Habbal et al, 2011 | AWD | *Aeromonas hydrophila,*  *Bacillus species,*  *Bacteriodes fragilis,*  *Citrobacter frewndii,*  *Clostridium perfringens*  *Corynebacterium diphtheriae,*  *Cryptococcus neoformans,*  *E. coli,*  *H. influenzae,*  *K. pneumoniae,*  *Micrococcus species,*  *N. meningitides,*  *P. aeruginosa,*  *S. aureus, S. epidermidis, MRSA,*  *Salmonella species,*  *Shigella sonnei,*  *Streptococcus pneumoniae,*  *Streptococcus pyogenes,*  *Vibrio cholerae,* | *Lawsonia inermis* L. (L) | *Lythraceae* | Methanol, ethanol and aqueous extracts | ZI |  |
| 76 | Nagarajan *et al*, 2013 | AWD | *"Bacillus subtitles, S. aureus, and Escherichia* | *Lawsonia inermis* L. (L) | *Lythraceae* | Ethanol, chloroform, hexane and methane | ZI |  |
| 77 | Maleki *et al*, 2018 | AWD and MTT MiD | *S. aureus ATCC 6538,*  *E. faecalis ATCC 1394,*  *P. aeruginosa ATCC 9027,*  *E. coli, ATCC 25922* | *Azadiractha Indica* L. (L) | *Meliaceae* | Ethanol, methanol, and ethyl acetate | ZI, MIC |  |
| 78 | Mohammed *et al*, 2018 | ADD | *E. faecalis*  *E. coli (21),*  *K. pneumoniae (21),*  *P. mirabilis (21),*  *P. aeruginosa (12),*  *S. aureus (17)* | *Azadiractha Indica* A.Juss. (L) | *Meliaceae* | 80 % Ethanol | ZI |  |
| 79 | Reddy *et al*, 2013 | AWD and MiD | *E. feacalis,*  *P. mirabilis*  *P. aeruginosa*  *S. aureus* | *Azadiractha Indica* A.Juss. (L, B, F) | *Meliaceae* | Aqueous | ZI |  |

* = measured at a population level or individual level; AD = Agar dilution; ADD = Agar disc diffusion; AWD = Agar well diffusion; BM = Broth microdilution; DD = Disc diffusion; MaD = Macrodilution; MIC = Minimum inhibitory concentrations; MiD = Microdilution; ZI = Zone of inhibition of bacterial growth; (L) = Leaves; (B) = Bark; (R) = Root; (SB)= Stem bark; (F) = Fruits; (Ae) = Aerial; (W) = Whole plant; (C) = Clove; (Bu) = Bulbs; (Ber) = berries; (Tu) = tuber; Fl = Flower; MRSA = Methicillin resistant *S. aureus*; VRE = Vancomycin resistant enterococcus.
