# Supplementary material for: Antibacterial and Antifungal Activities of Ethiopian Medicinal Plants: A Systematic Review
Source: Front Pharmacol. 2021 Jun 1;12:633921. doi: 10.3389/fphar.2021.633921 (PMC8203926; doi:10.3389/fphar.2021.633921)
Supplement: Supplementary file 2 [file Table2.docx]

Supplementary table 2: List of included studies for anti-fungal activities

| S/N | Studies | Name of the assay | Name of microorganisms (reference & clinical isolate) that medicinal plants activity conducted | Scientific name of the plant(s)/ compounds with their parts used | Family of the plants | Types of plants extracts/fractions /compounds | Outcome measured at a population level or individual level |
| --- | --- | --- | --- | --- | --- | --- | --- |
| **1** | Asres *et al*, 2006 | Microdilution method | *Candida albicans ATCC 10231, Aspergillus niger ATCC 6275, A. terreus MTCC 1782, Penicillium notatum ATCC 11625, P. funiculosum NCTC 287* and *P.citrinum MTCC 1256* | *Combretum molle* R.Br. ex G.Don (SB) | *Combretaceae* | Dichloromethane, acetone and methanol | Fungal growth MIC & MFC) |
| **2** | Sileshi *et a*l, 2007 | Agar well diffusion & Agar dilution method | *Trychophyton mentagrophytes (ATCC 18748), Candida albicans (clinical isolate), Aspergilus niger (ATCC10535),* and *A. flavus (ATCC 1397)* | *Clerodendrum myricoides (Hochst.) Steane & Mabb.* (L)*, Ficus plamata Forssk.* (L)*, Grewia ferruginea* Hochst. ex A. Rich. (L) *and Periploca linearifolia Quart. -Dill. & A. Rich.*  (Ae) | *Laminaceae, Moraceae, Tiliaceae, Asclepeiaceace* | Petroleum ether, chloroform, acetone and methanol | Zone of inhibition |
| **3** | Vaijayanthimala *et al*, 2000 | Microdilution method | *Candida albicans* | *Allium sativum* L. (C)*, Allium schoenoprasum L.* (C)*, Allium cepa var. cepa L* (Bu)*, Acalypha indica L* (L)*, Azedarach indica* A. Juss*.* (L &S)*, Camellia sinensis* (L.) Kuntze (L), *Cassia alata L.* (L)*, Cassia fstula L.* (L)*, Cassia occidentalis L (L), Coffea arabica L* (S)*, Curcuma longa L.*(R)*, Lawsonia inermis*  *L*.(L)*, Murraya koenigii* (L.) Spreng. *(*L)*, Ocimum sanctum L.* (L*), Piper betle L.* (L*), Psoralea corylifolia L.* (S) | *Liliaceae, Liliaceae, Liliaceae, Euphorbiaceae, Theaceae, Caesalpiniaceae, Caesalpiniaceae, Caesalpiniaceae, RubiaceaeZingiberaceae, Lythraceae, Rutaceae, Labiatae, Piperaceae, Papilionaceae* | Aqueous, 95% ethanol | Fungal growth (MIC and MFC) |
| **4** | Bora *et a*l, 2016 | Agar well diffusion & Microdilution method | *Candida albicans* | *Cinnamomum porrectum* (Jack) Meisn. (S)*, Lippia nudiflora* (L.) Greene (L), *Cestrum nocturnum L.* (F)*, Trachyspermum ammi* (L.) Sprague (S), *and Sida carpinifolia* L.f.  (F) | *Lauraceae Verbenaceae,‎ Solanaceae, Apiaceae, Malvaceae* | Methanol | Zone of inhibition & Fungal growth (MIC, MFC) |
| **5** | Park *et al*, 2007 | Agar well diffusion | *Dermatophytes Microsporum canis (KCTC 6591), Trichophyton mentagrophytes (KCTC 6077), Trichophyton rubrum (KCCM 60443), Epidermophyton floccosum (KCCM 11667),* and *Microsporum gypseum* | *Leptospermum petersonii*  F.M.Bailey (S), *Syzygium aromaticum*  (L.) Merr. & L.M.Perry (S) | *Myrtacea, Myrtaceae* | Essential oil | Zone of inhibition |
| **6** | Ameya *et al,* 2016 | Agar diffusion & agar dilution method | *C. albicans* and *A. flavus* | *Echinops kebericho* Mesfin (R) | *Asteraceae* | Ethanol, methanol and water | Zone of inhibition and Fungal growth (MIC & MFC) |
| **7** | Ameya *et al*, 2015 | Disc diffusion method, MIC | *clinical isolate of C. albicans* and *A. flavus* | *Taverniera abyssinica* A.Rich. (R) | *Fabaceae* | Ethanol, methanol and distilled water | Zone of inhibition, Fungal growth (MIC & MFC) |
| **8** | Gemeda *et a*l, 2014 | Agar dilution method & spore germination assay | *Aspergillus flavus (AF001, AF006, AF009, AF019, AF027,* and *AF037) and Aspergillus niger (AN002)* | *Cymbopogon martinii* (Roxb.) W.Watson (Ae)*, Foeniculum vulgar*  Mill*.* (Ae), *Trachyspermum ammi* (L.) Sprague (S) | *Poaceae, Apiaceae & Apiaceae* respectively | Essential oil | Zone of inhibition & Percent spore germination inhibition |
| **9** | Rana *et al,* 2011 | Agar well diffusion & Micro dilution method | *Fusarium moniliforme NCIM 1100, Fusarium oxysporum MTCC 284, Aspergillus species, Mucor species, Trichophyton rubrum* and *Microsporum gypseum* | *Syzygium aromaticum* (L.) Merr. & L.M.Perry (L.) | *Myrtaceae* | Essential oil | Zone of inhibition and Fungal growth (MIC & MFC) |
| **10** | Getie *et al*, 2003 | Agar well diffusion | *C. albicans, Aspergillus fumigatus* and *Trichophyton rubrum* | *Dodonaea viscosa* Jacq. (L)*; Rumex nervosus* (Engl.) Dammer (R)*; Rumex abyssinicus* Jacq. (R) | *Sapindaceae*  *Polygonaceae* | 80% Methanol | Zone of inhibition |
| **11** | Kasparaviciene *et al*, 2018 | Agar dilution method | *Candida albicans, richophyton spp. (nail and scalp isolated), Microsporum spp., Aspergillus niger* and *Rhodotorula rubra* | *Oleogel* | *Lamiaceae* | Essential oil | Zone of inhibition |
| **12** | Fierascu *et al*, 2018 | Agar well diffusion | *Aspergillus niger ATCC 15475 and Penicillium hirsutum ATCC 52323* | *Juniperus communis* L. (F) | *Cupressaceae* | 50% Ethanol | Zone of inhibition |
| **13** | Nyanchok, 2007 | Agar well diffusion | *Candida albicans, Penicillium notatum* | *Bersama abyssinica* Fresen. (SB) | *Francoaceae* | N-hexane, dichlomethane, ethyl acetate and methanol | Zone of inhibition |
| **14** | Jain *et al,* 2017 | Macro-dilution (Tube dilution) method | *Trichophyton rubrum (MTCC 296), T. mentagrophytes (MTCC 7687), Microsporum gypseum (MTCC 4524), M. fulvum(MTCC2837), T. soudanense* and *T. interdigitale* | *Thymus vulgaris* L. (L) | *Lamiaceae* | Essential oil | Fungal growth (MIC & MFC) |
| **15** | Messele *et al*, 2004 | Agar well diffusion | *T. mentagrophytes (ATCC), A. niger (ATCC)* and *C. albicans (isol.)* | *Inula confertiflora* A. Rich. (L, F)*, Clematis simensis* Fresen. (L)*, Zehneria scabra* (L.f.) Sond. (L)*, Pycnostachys abyssinica* Fresen. *(*L)*,* | *Compositae, Ranunculaceae,*  *Cucurbitacea* & *Labiatae respectivelt* | Petroleum ether, 80% methanol | Zone of inhibition |
| **16** | Salazar *et al*, 2015 | Broth microdilution method | *Trichophyton menta, Trichophyton rubrum, Epidermophyton floccos, Microsporum canis* | *Azadirachta indica* A.Juss. (L) | *Meliaceae* | Oil from seed | Fungal growth (MIC) |
| **17** | Simhadri *et al*, 2017 | Disc diffusion & Microdilution | *Trichophyton rubrum (MTCC7859), Microsporum gypseum (MTCC 4524), Epidermophyton floccosum (MTCC 7880)* | *Azadirachta indica* A.Juss. (L) | *Meliaceae* | Hexane, benzene, ethyl acetate, methanol | Zone of inhibition and Fungal growth (MIC) |

Leaves = L, Bark =B, Root=R, Stem bark=SB, Fruits=F, Aerial =Ae, Whole plant=W, Clove=C, Bulbs=Bu, Minimum inhibitory concentrations = MIC, minimum fungicidal concentration =MFC.
