# Supplementary material for: Antibacterial and Antifungal Activities of Ethiopian Medicinal Plants: A Systematic Review
Source: Front Pharmacol. 2021 Jun 1;12:633921. doi: 10.3389/fphar.2021.633921 (PMC8203926; doi:10.3389/fphar.2021.633921)
Supplement: Supplementary file 3 [file Table3.docx]

**Supplementary Table 3- Botanical or multiherbal for antibacterial studies**

| **Study** | **Species, sources, concentrations** | **Quality control reported (Y/N)** | **Chemical analysis reported (Y/N)** |
| --- | --- | --- | --- |
| Oumer et al (2014) | - Leaf latex of *Aloe trichosantha A.Berger,* collected from its habitat, 5 to 800 μg/mL | Y – extracts prepared as per the protocol reported previously | Y - PTLC |
| Amoo et al (2012) | - Stem, root and whole plant of *Huernia hystrix (Hook.f.) N.E. Br,* collected from its habitat*,* 0.2mg/mL | Y – extracts prepared as per the protocol reported previously | Y- qualitative phytochemical screening was performed |
| Techana et al (2012) | - Leaves and roots *Entada abyssinica* Steud. ex A. Rich.*,* collected from its habitat, 64-024 µg/mL - Bark *Entada africana* Guill. & Perr., collected from its habitat, 64-1024 µg/mL - Bark of *Carica papaya L,* collected from its habitat*,* 64-1024 µg/mL - Seeds of *Carica papaya L,* collected from its habitat*,* 64-1024 µg/mL - Stones of *Persea americana Mill,* collected from its habitat, 64 -1024 µg/mL | Y- extracts were prepared as per the protocol reported previously | N |
| Romha et al (2017) | - Aerial part of *Calpurnia aurea (Aiton) Benth.,* collected from its habitat, 200, 100, and 50mg/ml - Leaves of *Croton macrostachyus Hochst. ex Delile,* collected from its habitat, 200, 100, and 50mg/ml - Leaves of *Withania somnifera (L.) Dunal,* collected from its habitat, 200, 100, and 50mg/ml | Y- plant extracts were collected, identified, extracted and tested as per previously mentioned protocols | N |
| Taye et al (2011) | - Leaves of *Achyranthes aspera L*, 500mg/ml - Roots of *Brucea antidysenterica J.F.Mill.*, 500mg/ml - Leaves of *Datura stramonium L.*, 500mg/ml - Leaves of *Croton macrostachyus Hochst. ex Delile,* 500mg/ml - Leaves of *Acokanthera schimperi (A.DC.) Schweinf.*, 500mg/ml - *Roots of Phytolacca dodecandra L’Hér.*, 500mg/ml - Leaves of *Millettia ferruginea (Hochst.) Hochst. ex Baker,*500mg/ml - Leaves of *Solanum incanum L.,* 500mg/ml | Y- plant extracts were collected, identified, extracted and tested as per previously mentioned protocols | N |
| Belal et al (2017) | - Seed of *Cuminum cyminum L..*, purchased from local market, 12.5%, 25%, 50% & 100% | Y – the oil of *Cumin cyminum* extracted and tested using previously used method | Y – preliminary phytochemicals analysis was performed |
| Baynesagne et al (2017) | - Leaves of *Datura stramonium* L., collected from different regions of Gondar town, Ethiopia, 50 mg/ml | Y-extracts were extracted, prepared and tested using previously mentioned methods (Taye et al., 2011) | Y – preliminary phytochemicals analysis was performed |
| Vazirian et al (2016) | - Seeds of *Trachyspermum ammi (L.) Sprague (L.) Sprague,* purchased from local medicinal plant vender, 100-0.02 µL/well | Y – Oil of *Trachyspermum ammi* was extracted and tested using methods reported previously. | Y – preliminary phytochemical analysis using GC-MS method |
| Yeabyo et al (2018) | - The roots of *Verbascum erianthum Benth.,* collected from Tigray region, Ethiopia, 1 mg/ml | Y - the roots of *Verbascum sinaiticum* were dried, ground, extracted and tested using previously reported method | Y- preliminary phytochemical analysis |
| Begashawu et al (2016) | - Leaves of *Kosteletzkya begonifolia (Ulbr.) Ulbr,* collected from its natural habitat, 0.2, 0.1 and 0.05 g - Leaves of *Leucas martinicensis (Jacq.) R.Br.,* collected from its natural habitat, 0.2, 0.1 and 0.05 g - Leaves of *Ranunculus multifidus Forssk.,* collected from its natural habitat, 0.2, 0.1 and 0.05 g | Y- Plant materials were, extracted and tested using previously used method | Y-preliminary photochemical screening was performed |
| Burt et al (2003) | - Leaves of *Pimenta racemosa (Mill.) J.W.Moore*, purchased from C. Melchers Essential Oils Handels-GmbH, Germany; 78, 156, 312 and 625 µl/l - Leaves of *Eugenia caryophyllata Thunberg*, Purchased from C. Melchers Essential Oils Handels-GmbH, Bremen, Germany; 78, 156, 312 and 625 µl/l - Leaves of *Origanum vulgare* L.*,* Purchased from C. Melchers Essential Oils Handels-GmbH, Bremen, Germany; 78, 156, 312 and 625 µl/l - Leaves of *Thymus vulgaris* L.*,* Purchased from C. Melchers Essential Oils Handels-GmbH, Bremen, Germany; 78, 156, 312 and 625 µl/l | Y- tests were performed as per previously mentioned method | N |
| Bisht et al (2014) | - Leaves of *Withania somnifera (L.) Dunal,* collected from the Central Institute of Medicinal and Aromatic Plants, India, 1mg/ml and 2mg/ml | Y- extraction and tests were performed according to Owais et al (2005) and Janssen et al. (1987). | N |
| Djeussi et al (2016) | - Fruit, leaves and bark of *Anthocleista schweinfurthii* Gilg, collected from South West regions of Cameroon ,128 to 1024 μg/mL - Fruit, leaves and bark *Nauclea latifolia Sm.*, collected from South West regions of Cameroon, 128 to 1024 μg/mL - Whole plant of  *Boehmeria virgata var. macrostachya (Wight) Friis & Wilmot-Dear*, collected from South West regions of Cameroon, 128 to 1024 μg/mL - Whole plant of *Caucalis melanantha (Hochst.) Benth. & Hook.f. ex Hiern*, collected from South West regions of Cameroon, 128 to 1024 μg/mL - Whole plant of *Erigeron floribundus (Kunth) Sch.Bip.*, collected from South West regions of Cameroon, 128 to 1024 μg/mL - Whole plant of *Zehneria scabra (L.f.) Sond.,* collected from South West regions of Cameroon, 128 to 1024 μg/mL | Y- plant materials were dried, ground, extracted and tested using previously reported methods | Y-preliminary photochemical screening was performed |
| Asres et al (2006) | - Bark of *Combretum molle R.Br. ex G.Don,* collected from Gual Mereb in Tigray region, Ethiopia, 200 mg/ ml | Y- extraction using Soxhlet apparatus and extracts were tested using previously reported methods | N |
| Sileshi et al (2008) | - Leaves of *Rotheca myricoides (Hochst.) Steane & Mabb.*, collected from their natural habitats in Ethiopia, 50 mg/ml - Leaves of *Ficus palmata Forssk.*, collected from natural habitat in Ethiopia, 50 mg/ml - Leaves of *Grewia ferruginea Hochst. ex A.Rich.* - *,* Collected from the natural habitat in Ethiopia, 50 mg/ml - Aerial part of *Periploca linearifolia Quart. -Dill. & A.Rich.*, Collected from the natural habitat in Ethiopia, 50 mg/ml | Y- plant materials were extracted and tested using previously mentioned methods | N |
| Habtamu et al (2018) | - Leaves of *Vernonia amygdalina Delile ,* collected from Wonji district, 10 mg/mL | Y – plant material was extracted successively and pharmacological activities were tested using previously reported method | Y – TLC, NMR, UV and IR |
| Adedapo et al (2008) | - Stem and leaves of *Calpurnia aurea (Aiton) Benth.,* collected from Eastern Cape Province of South Africa, 0.1- 5 mg/ml. | Y- plant material extraction and pharmacological activity tests were performed as per the | Y- quantitative phytochemical screening was performed |
| Habtamu et al (2017) | - Leaves of *Clematis hirsuta Guill. & Perr.,* collected from natural habitat in Ethiopia; 50, 25, 12.5, 6.75 and 3.125 mg/ml | Y - The plant material was extracted and tested for antibacterial activity using methods reported previously | N |
| Dua et al (2013) | - Stem *Cuminum cyminum L.,* procured from the local market in India, 100 µl/well | Y- extraction of the essential and test against bacteria was done according to previously reported methods | N |
| Umer et al (2013) | - Leaves of *Calpurnia aurea (Aiton) Benth.,* collected from Addis Ababa, Ethiopia, 250 mg/ml | Y- extraction and antibacterial activity was performed according to methods previously reported | Y - Preliminary phytochemical screening was performed |
| Vijayasanthi et al (2014) | - Leaves of *Delonix elata (L.) Gamble,* collected from its natural habitat in India, 100 &200µg/ml - Leaves of *Spathodea campanulata P.Beauv.,* collected from its habitat in India, 100 &200µg/ml | Y – extraction and antibacterial testes were performed as per methods reported before. | N |
| Tadeg et al (2005) | - Leaves of *Acokanthera schimperi (A.DC.) Schweinf.*, collected from its habitat in Ethiopia, 100, 50 and 25 mg/ml - Leaves of *Calpurnia aurea (Aiton) Benth.*, collected from its habitat in Ethiopia, 100, 50 and 25 mg/ml - Leaves of *Kalanchoe petitiana A.Rich.* - , collected from its habitat in Ethiopia, 100, 50 and 25 mg/ml - Leaves of *Lippia abyssinica (Otto & A.Dietr.) Cufod.*, collected from its habitat in Ethiopia, 100, 50 and 25 mg/ml - Leaves of *Olinia rochetiana A.Juss.*, collected from its habitat, 100, 50 and 25 mg/ml - Leaves of *Verbascum erianthum Benth.*, collected form its habitat in Ethiopia, 100, 50 and 25 mg/ml - Fruit of *Phytolacca dodecandra L'Hér.,* collected from its habitat in Ethiopia, 100, 50 and 25 mg/ml - Roots of *Malva parviflora L.,* collected from its habitat in Ethiopia, 100, 50 and 25 mg/ml. | Y - Plant materials extraction, fractionation and antimicrobial tests were performed as per previously report method | N |
| Mwitari et al (2013) | - Aerial of *Withania somnifera (L.) Dunal.,* collected from its natural habitat in Kenya, 100 mg/ml - Stem bark of *Prunus africana (Hook.f.) Kalkman,* collected from its natural habitat in Kenya, 100 mg/ml - Stem bark of *Warburgia ugandensis Sprague,* collected from its natural habitat in Kenya, 100 mg/ml - Stem bark of *Plectranthus glandulosus Hook.f.* - *,* collected from its natural habitat in Kenya, 100 mg/ml | Y – extraction and antibacterial tests were done as per methods reported previously | N |
| Seshathri *et al* (2011) | - Stem of *Clausena anisata (Willd.) Hook.f. ex Benth.*, collected from its habitat in Ethiopia, 25-100mg/ml - Stem of *Clematis simensis Fresen.*, collected from its habitat in Ethiopia, 25-100mg/ml - Stem of *Rotheca myricoides (Hochst.) Steane & Mabb.*, collected from its habitat in Ethiopia, 25-100mg/ml - Stem of *Juniperus procera Hochst. ex Endl.*, collected from it habitat in Ethiopia, 25-100mg/ml - Stem of *Justicia schimperiana T.Anderson* - , collected from its habitat in Ethiopia, 25-100mg/ml - Stem of *Olea europaea L.*, 25 -collected from its habitat in Ethiopia, 100mg/ml - Stem of *Phoenix reclinata Jacq.* (Petiole), collected from its habitat in Ethiopia, 25-100mg/ml - Stem of  *Rubus apetalus Poir..*, collected from its habitat in Ethiopia, 25-100mg/ml - Stem of *Sesbania sesban (L.) Merr.*, collected from its habitat in Ethiopia, 25 -100mg/ml - Stem of *Sida rhombifolia L.*, collected from its habitat in Ethiopia, 25-100mg/ml - Flower of *Acmella caulirhiza Delile*, collected from its habitat in Ethiopia, 25-100mg/ml - Stem of *Stereospermum kunthianum Cham.,* collected from its habitat in Ethiopia, 25-100mg/ml - Stem of *Gymnanthemum amygdalinum (Delile) Sch.Bip.,* collected from its habitat in Ethiopia, 25-100mg/ml | Y - plant materials extraction and antibacterial tests were as per methods reported before. | N |
| Obey *et al,* 2016 | - Stem bark of *Croton macrostachyus Hochst. ex Delile,* from its habitat in Kenya, 1.9 -500 mg/ml | Y - plant materials were extracted using pervious methods, and antibacterial activity was conducted according to Taye et al (2011) | Y- NMR |
| Ewansiha *et al,* 2012 | - Leaves of *Cymbopogon citratus (DC.) Stapf.*, collected from its habitat in Nigeria, | Y - plant material extraction and antibacterial activity tests were done using methods previously reported. | Y - preliminary phytochemical screening was performed |
| Ameya *et a*l, 2016 | - Roots of *Echinops kebericho*Mesfin*,* collected from its habitat in Ethiopia, 200 µg/ml, 1.56-100µg/ml | Y - plant material was extracted and antibacterial were tested | N |
| Singh *et al,* 2011 | - Oil of *Cymbopogon citratus (DC.) Stapf,* obtained from Naga Fragrance Pvt. Ltd, India, 1024 µg /ml, 1-256 µg /ml | Y- Antibacterial activity was tested using methods previously reported | N |
| Duraipandiyan et al (2012) | - Rhizomes *Hellenia speciosa (J.Koenig) S.R.Dutta,* collected from its natural habitat in India, 5, 2.5 and 1.25 mg per disc | Y - extraction, fractionation and compound isolation | Y- GC-MS |
| Ameya et al (2015) | - Root of *Taverniera abyssinica A.Rich.*, purchased from local market in Addis Ababa, 10, 20, 40 and 80 mg/mL | Y - extraction, and antibacterial activity tests were performed using methods reported before | N |
| Hassanshahian et al (2014) | - Seed of *Trachyspermum ammi (L.) Sprague,* collected from Iran, 250ppm, 100ppm, 50ppm and 10ppm | Y – essential oil extraction and antibacterial test were performed as per methods used previously | N |
| Lulekal et al (2014) | - Leaves of *Bersama abyssinica Fresen.*, collected from its habitat in Ethiopia, 51.2 mg/ml, - Root of *Calpurnia aurea (Lam.) Benth*, collected from its habitat in Ethiopia, 51.2 mg/ml - Roots of *Carissa spinarum L.*, collected from its habitat in Ethiopia, 51.2 mg/ml - Leaves of *Clematis hirsuta Guill. & Perr.*, collected from its habitat in Ethiopia, 51.2 mg/ml - Roots of *Clutia abyssinica Jaub. & Spach*, collected from its habitat in Ethiopia, 51.2 mg/ml - Leaves of *Croton macrostachyus Hochst. ex Delile.*, collected from its habitat in Ethiopia, 51.2 mg/ml - Roots of *Cyathula cylindrica Moq.*, collected from its habitat in Ethiopia, 51.2 mg/ml - Leaves of *Dodonaea viscosa subsp. angustifolia (L.f.) J.G.West.*, collected from its habitat in Ethiopia, 51.2 mg/ml - Stem of *Embelia schimperi Vatke*, collected from its habitat in Ethiopia, 51.2 mg/ml - Leaves *Jasminum abyssinicum Hochst. ex DC.*, collected from its habitat in Ethiopia, 51.2 mg/ml - Leaves of *Maesa lanceolata Forssk*, collected from its habitat in Ethiopia, 51.2 mg/ml - Leaves of *Ocimum lamiifolium Hochst. ex Benth.*, collected from its habitat in Ethiopia, 51.2 mg/ml - Leaves of *Olinia rochetiana A.Juss.*, collected from its habitat in Ethiopia, 51.2 mg/ml - Roots of Rubus *Rubus steudneri Schweinf.*, collected from its habitat in Ethiopia, 51.2 mg/ml - Roots of *Rumex nepalensis Spreng*, collected from its habitat in Ethiopia, 51.2 mg/ml - Roots of *Thalictrum rhynchocarpum Quart. -Dill. & A.Rich..*, collected from its habitat in Ethiopia, 51.2 mg/ml - Leaves of *Verbascum erianthum Benth.*, collected from its habitat in Ethiopia, 51.2 mg/ml - Fruit of *Gymnanthemum amygdalinum* (Delile) Sch.Bip., collected from its habitat in Ethiopia, 51.2 mg/ml | Y - plant material extraction and antibacterial activity tests were done according to previously reported methods | N |
| Bacha et al, 2016 | - Fruit of *Aframomum corrorima (A.Braun) P.C.M.Jansen,* collected from its habitat in Ethiopia, 500mg/ml - Root of *Albizia schimperiana Oliv.,* collected from its habitat in Ethiopia, 500mg/ml - Rhizome of *Curcuma longa L.,* collected from its habitat in Ethiopia, 500mg/ml - Stem bark of *Erythrina brucei Schweinf. emend. Gillett.,* collected from its habitat in Ethiopia, 500mg/ml - Stem of *Justicia schimperiana (Hochst. ex Nees) T. Anderson* - *,* collected from its habitat in Ethiopia, 500mg/ml - Stem leaves of *Nigella sativa L.,* collected from its habitat in Ethiopia, 500mg/ml - Leaves of *Ocimum gratissimum subsp. gratissimum.,* collected from its habitat in Ethiopia, 500mg/ml - Leaves of *Gymnanthemum amygdalinum (Delile) Sch.Bip.,* collected from its habitat in Ethiopia, 500mg/ml | Y – extraction and antibacterial activity were performed according to methods reported previously | N |
| Njeru et al, 2015 | - Roots of *Premna resinosa (Hochst.) Schauer,* collected from its habitat in Kenya, 500, 250 μg/ml | Y – extraction and antibacterial activity was performed as per previously reported protocol | N |
| Ngeny et al (2013) | - Leaves and stem bark of *Hagenia abyssinica (Bruce) J.F.Gmel.*, collected from its habitat in Kenya, 100 mg/ml - Aerial parts of *Fuerstia africana T.C.E.Fr..,* collected from itshabita in Kenya, 100mg/ml - Roots of *Ekebergia capensis Sparrm,* collected from its habitat in Kenya, 100mg/ml - Stem bark of *Asparagus racemosus Willd..,* collected from its habitat in Kenya, *100*mg/ml | Y – plant materials extraction and antibacterial activity tested were performed as per previously reported protocols | Y – preliminary phytochemical screening was performed |
| Hussien et al (2011) | - Stem bar of *Brassica nigra (L.) W.D.J.Koch*, collected from Jima area, Ethiopia, 5, 10, 15% - Leaves of *Thymus schimperi Ronniger* - , collected from Jima area, Ethiopia, 5, 10, 15% - Leaves of *Ocimum basilicum L.*, collected from Jima area, Ethiopia, 5, 10, 15% - Fruit of *Syzygium aromaticum (L.) Merr. & L.M.Perry*, collected from Jima area, Ethiopia, 5, 10, 15% - Fruit of *Elettaria cardamomum (L.) Maton.,* collected from Jima area, Ethiopia, 5, 10, 15% - Stem bark of *Cinnamomum verum J.Presl,* collected from Jima area, Ethiopia, 5, 10, 15% | Y - plant material preparation and antibacterial activity tests were done using method previously reported | N |
| Debalke et al (2018) | - Whole plant parts *Sida rhombifolia L.,* collected from its habitat in Ethiopia, 500mg/ml, 250mg/ml, 125mg/ml, and 62.5mg/ml | Y - plant material extractions, antibacterial activities were done as per previously reported methods | Y - Preliminary phytochemical screening |
| Unnithan et al (2013) | - Aerial part of *Ocimum basilicum L..*, collected from its habitat, 20mg/ml | Y – Essential oil extraction and antibacterial activity tests were done as per the protocols reported before | Y - GC and GC-MS |
| Meshesha et al (2017) | - Leaves of *Kniphofia uvaria (L.) Oken.,* collected from its habitat in Ethiopia, 50 mg/mL | Y - Extraction, isolation and antibacterial activity was performed using methods previously reported | Y - NMR |
| Gadisa et al (2019) | - Leaves of *Blepharis cuspidata Lindau.,* collected from its habitat in Ethiopia, 100µl/ml - Leaves of *Boswellia ogadensis Vollesen* - *,* collected from its habitat in Ethiopia, 100µl/ml - Leaves of *Thymus schimperi Ronniger* - *.,* collected from its habitat in Ethiopia, 100µl/ml | Y - essential oil extractions and tests for antibacterial activities were performed as per the protocol mentioned before. | N |
| Belay et al (2011) | - Leaves of *Artemisia absinthium*L*.* - *,* collected from its habitat, 50 to 0.1 μl/ml - Leaves of *Artemesia abyssinica, ,* collected from its habitat, 50 to 0.1 μl/ml - Leaves of *Croton macrostachyus Hochst. ex Delile*, collected from its habitat, 50 to 0.1 μl/ml - Tuber of *Echnops kebericho* Mesfin*,* collected from its habitat in Ethiopia, *50* to 0.1 μl/ml - Berries of *Satureja punctata (Benth.) R.Br. ex Briq.* - *,* collected from its habitat in Ethiopia, 50 to 0.1 μl/ml | Y – Essential oils extraction and anti-bacterial activity tests were performed as per methods previously reported | N |
| Mulat et al (2015) | - Leaves of *Ocimum gratissimum subsp. gratissimum.*, collected from its habitats in Jima area, Ethiopia, 0.08mg/ml-20mg/ml - Leaves of  *Ruta graveolens L..*, collected from its habitat in Jima area, Ethiopia, 0.08mg/ml-20mg/ml - Leaves of *Ocimum lamiifolium Hochst. ex Benth..,* 0.08mg/ml-20mg/ml - Seeds of *Nigella sativa* L., collected from its habitat in Jima area, Ethiopia, 0.08mg/ml-20mg/ml | Y – extraction of plant materials and essential oils, | Y - preliminary phytochemical screening was performed |
| Chalo et al (2015) | - Bark of *Schrebera alata (Hochst.) Welw*, collected from its natural habitat in Kenya, 400, 200, 100 mg/ml - Aerial part of *Ormocarpum kirkii S.Moore*, collected from its natural habitat in Kenya, 400, 200, 100 mg/ml - Bark of *Cussonia holstii Harms ex Engl.,* collected from its natural habitat in Kenya, 400, 200, 100 mg/ml - Whole plant of *Helichrysum forskahlii (J.F.Gmel.) Hilliard & B.L.Burtt.,* collected from its natural habitat in Kenya, 400, 200, 100 mg/ml | Y - plant materials extraction and anti- bacterial activity was performed using methods previously mentioned | Y- preliminary phytochemical screening was performed |
| Habtamu et al (2017) | Leaves of *Achyranthes aspera L.*, collected from the natural vegetation in Ethiopia, 50, 100 and 200 mg/ml | Y – plant material extraction and antibacterial activity test were performed using methods previously reported | N |
| Genanew et al (2017) | - Leaves of *Aloe macrocarpa Tod*, collected from its natural habitat in Ethiopia, 2, 1 and 0.5 mg/ml, 0.625–20 μg/ml | Y – Plant material collection and antibacterial activity testes were done using methods previously reported | Y - Preliminary phytochemical analysis was performed |
| Hagos et al (2017) | - Leaves of *Moringa stenopetala (Baker f.) Cufod..*, collected from its natural habitat in Ethiopia, 50 mg/ml | Y – plant materials extraction and antibacterial tests were performed according to protocols reported before. | N |
| Ameya et al (2018) | - Leaves of *Nicotiana tabacum L.,* collected from its natural habitat in Ethiopia, 1 mg/ml | Y – plant material extraction and antibacterial activities were performed according to methods reported before. | Y - GC - MS |
| Abew et al (2014) | - Leaves of *Zehneria scabra (L.f.) Sond.*, collected from its natural habitat in Ethiopia, 100 mg/ mL, 200 mg/mL, 300 mg/mL, 400 mg/mL and 500 mg/mL - Leaves of *Ricinus communis L..,* collected from its natural habitat in Ethiopia | Y – plant materials were extracted and tested using methods previously reported | N |
| Mummed et al (2018) | - Aerial of  *Cissus quadrangularis L.*, collected from its natural habitat in Ethiopia, 780,390, and 195 mg/mL - Leaves of *Commelina benghalensis L.*, collected from its natural habitat in Ethiopia, 780,390, and 195 mg/mL - Roots of *Euphorbia heterophylla L.*, collected from its natural habitat in Ethiopia, 780,390, and 195 mg/mL - Whole plant of  *Euphorbia prostrata Aiton*, collected from its natural habitat in Ethiopia, 780,390, and 195 mg/mL - Leaves of *Grewia villosa Willd.*, collected from its natural habitat in Ethiopia, 780,390, and 195 mg/mL - Fruit of *Momordica foetida Schumach.*, collected from its natural habitat in Ethiopia, 780,390, and 195 mg/mL - Aerial of *Trianthema portulacastrum L.*, collected from its natural habitat in Ethiopia, 780,390, and 195 mg/mL - Leaves of *Schinus molle L.,* collected from its natural habitat in Ethiopia, 780,390, and 195 mg/mL - Fruit of *Solanum incanum L.,* collected from its natural habitat in Ethiopia, 780,390, and 195 mg/mL | Y – plant materials extraction and antibacterial activity tests were performed according to previously reported methods | Y – preliminary phytochemical screen was performed |
| Minale et al (2014) | - Leaves of *Aloe sinana Reynolds*, collected from its natural habita in Ethiopia, 200 µg/ml | Y – plant materials were extracted and antibacterial tests were performed | PTLC, NMR |
| Moglad et al (2014) | - Leaves and stems of *Maerua oblongifolia (Forssk.) A.Rich.*, collected from its natural habitat in Sudan, 2.5, 5, 10 and 20 mg\mL | Y - plant materials were extracted and antibacterial activity was performed using standard protocols | Y - preliminary phytochemicals analysis was performed |
| Hawaze et al (2012) | - Leaves of *Clematis longicauda Steud. ex A.Rich.*, collected from its natural habitat in Ethiopia, 250, 500 and 1000μg/ml, - Leaves of *Clematis hirsuta Guill. & Perr.*, collected from its natural environment in Ethiopia, 250, 500 and 1000 μg/ml | Y – plant material extractions and antibacterial activity tests were performed using standard protocols | Y -preliminary phytochemical analysis was performed |
| Regassa et al (2012) | - Leaves, bark, stem and seed Combretum molle R.Br. ex G. Don, collected from its natural habitat in Ethiopia, 100-mg/ml solution. | Y – plant material extractions and antibacterial activity tests were performed using standard protocols | N |
| Zulfa et al (2015) | - Leaves of *Cymbopogon citratus (DC.) Stapf*, collected from garden in Malaysia, 100 mg/ml, 10 mg/ml | Y- essential oil extraction and antibacterial activities were performed as per protocols reported before | N |
| Ameya et al (2018) | - Fruit of *Capsicum frutescens L.,* purchased from local market in Ethiopia, 0.1gm/ml, 100mg/L to 12.5mg/L | Y – plant material extraction and antibacterial activity tests were performed standard protocols | Y – preliminary phytochemical screening was performed |
| Teka et al (2015) | - Stem bark *Apodytes dimidiata E.Mey. ex Arn.*collected from its natural habitat in Ethiopia, 4–512 μg/ml - Leaves of *Asparagus africanus Lam.*, collected from its natural habitat in Ethiopia, 4–512 μg/ml - Stem of *Bersama abyssinica Fresen.,* collected from its natural habitat in Ethiopia, 4–512 μg/ml - Roots of *Cucumis ficifolius A.Rich.,* collected from its natural habitat in Ethiopia, 4–512 μg/ml - Bulbes of *Gladiolus abyssinicus (Brongn. ex Lem.) B.D.Jacks..* collected from its natural habitat in Ethiopia, 4–512 μg/ml - Leaves of *Guizotia schimperi Sch.Bip.,* collected from its natural habitat in Ethiopia, 4–512 μg/ml - Leaves of *Lippia abyssinica (Otto & A.Dietr.) Cufod.*, collected from its natural habitat in Ethiopia, 4–512 μg/ml - Stem bark of *Olinia rochetiana A.Juss.*, collected from its natural habitat in Ethiopia, 4–512 μg/ml - Leaves of *Pavonia urens Cav.*, collected from its natural habitat in Ethiopia, 4–512 μg/ml - Leaves of *Premna schimperi Engl.* , collected from its natural habitat in Ethiopia, 4–512 μg/ml - Leaves of *Pittosporum viridiflorum Sims* , collected from its natural habitat in Ethiopia, 4–512 μg/ml - Roots of *Polygala sadebeckiana Gürke*, collected from its natural habitat in Ethiopia, 4–512 μg/ml - Roots of *Sida rhombifolia L.*, collected from its natural habitat in Ethiopia, 4–512 μg/ml - Fruit of ***Solanum incanum var. integrascens Bitter****,* collected from its natural habitat in Ethiopia, 4–512 μg/ml | Y – plant material extraction and antibacterial activity tests were performed standard protocols | Y – preliminary phytochemical screening was performed |
| Asamenew et al (2011) | - Leaves of *Aloe harlana Reynolds,* collected from its natural habitat in Ethiopia, 200µg/mL, 5 to 800 µg/mL | Y - Extraction, compound isolation and antibacterial activity tests were done using methods previously reported | Y - NMR |
| Kalayou et al (2012) | - Leaves of *Ficus carica L.,* collected from its environmental habitat in Ethiopia, 200 mg/mL and100 mg/mL, 6.25 to 200 mg/mL - Leaves of *Malva parviflora L.*, collected from its environmental habitat in Ethiopia, 200 mg/mL and100 mg/mL, 6.25 to 200 mg/mL - Leaves of *Gymnanthemum amygdalinum (Delile) Sch.Bip.*, collected from its environmental habitat in Ethiopia, 200 mg/mL and100 mg/mL, 6.25 to 200 mg/mL - Leaves of *Solanum hastifolium Hochst. ex Dunal*, collected from its environmental habitat in Ethiopia, 200 mg/mL and100 mg/mL, 6.25 to 200 mg/mL - Leaves of *Calpurnia aurea (Aiton) Benth.,* collected from its environmental habitat in Ethiopia, 200 mg/mL and100 mg/mL, 6.25 to 200 mg/mL - Leaves of *Nicotiana tabacum L.,* collected from its environmental habitat in Ethiopia, 200 mg/mL and100 mg/mL, 6.25 to 200 mg/mL - Leaves and stem of *Ziziphus spina-christi (L.) Desf.,* collected from its environmental habitat in Ethiopia, 200 mg/mL and100 mg/mL, 6.25 to 200 mg/mL - Leaves of *Croton macrostachyus Hochst. ex Delile,* collected from its environmental habitat in Ethiopia, 200 mg/mL and100 mg/mL, 6.25 to 200 mg/mL | Y – Extraction and antibacterial activity tests were done using methods previously reported | Y- preliminary phytochemical screening was performed |
| Begashaw et al (2017) | - Leaves of *Hibiscus micranthus L.f.,* collected from its environmental habitat in Ethiopia, 200, 400 and 800 μg/ml | Y – Extraction and antibacterial activity tests were done using methods previously reported | Y- preliminary phytochemical screening was performed |
| Bekele et al (2015) | - Leaves of *Thymus schimperi Ronniger* - *,* purchased from local market in Addis Ababa, Ethiopia, 50mg/ml, 3.12-50mg/ml | Y - plant material extraction and antibacterial activity tests was done using standard methods reported before | N |
| Palla et al (2015) | - Stem of *Linum usitatissimum subsp. intermedium Chernom.* - *,* purchased from authenticated herb supplier in Pakistan, 180 μg/μL, 100, 300 and 500 mg/kg | Y – Flaxseed oil extraction and antibacterial activity tests were done using standard methods reported previously | N |
| Albejo et al (2015) | - Leaves of *Vernonia auriculifera Hiern* - *,* collected from its natural habitat in Ethiopia, 0.8-400mg/ml | Y – extraction and antibacterial activity test were done as per standard methods reported before. | Y – preliminary phytochemical screening was performed |
| Abdissa et al (2015) | - Leaves of *Aloe pulcherrima M.G.Gilbert & Sebsebe,* collected from its natural habitat in Ethiopia, 50 mg/mL | Y – sequential extraction of plant material and antibacterial activity was performed using methods reported before. | Y – Column chromatography |
| Girmay et a (2017) | - Seed of *Lepidium sativum L.,* collected from its natural habitat in Ethiopia, 10 µL and 20 µL per well | Y – extraction and antibacterial activity test were done as per standard methods reported before. | Y – preliminary phytochemical screening was performed |
| Seid (2016) | - Leaves of *Foeniculum vulgare Mill.,* collected from its natural habitat in Ethiopia, 0.2 g /mL | Y – extraction and antibacterial activity test were done as per standard methods reported before. | Y – preliminary phytochemical screening was performed, GC-MS |
| Teshome et al (2018) | - Leaves of  *Clematis simensis Fresen.,* collected from its natural habitat, 1,2,3 4 and 5mg/ml | Y – extraction and antibacterial activity test were done as per standard methods reported before. | Y – preliminary phytochemical screening was performed |
| Girmay et al (2015) | - Leaves of *Datura stramonium L.,* collected from its natural habitat, 20 and 40 µg/m | Y – extraction and antibacterial activity test were done as per standard methods reported before. | Y – preliminary phytochemical screening was performed |
| Nyanchoka (2016) | - Stem bark of *Bersama abyssinica Fresen.,* collected from its natural habitat in Kenya, 500µg/disc | Y – extraction, fractionation, compound isolation and anti-bacterial activities were done according to methods previously reported | NMR, HPLC |
| Goji et al (2006) | - Leaves of *Jasminum abyssinicum Hochst. ex DC.*, collected from its natural habitat, 25 mg/ml, 50 mg/ml and 100 mg/ml - Leaves of *Solanecio gigas (Vatke) C.Jeffrey*, collected from its natural habitat, 25 mg/ml, 50 mg/ml and 100 mg/ml - Leaves, seeds and fruits of *Lagenaria siceraria (Molina) Standl.*, collected from its natural habitat, 25 mg/ml, 50 mg/ml and 100 mg/ml | Y – extraction, fractionation and antibacterial activity test were done as per standard methods reported before | N |
| Megeressa et al (2015) | - Leaves of *Aloe trigonantha L.C.Leach ,* collected from its natural habitat, 25 mg/ml, 50 mg/ml and 100 mg/ml | Y - extraction, fractionation, compound isolation and antibacterial activities were performed methods reported before | Y - HPLC |
| Hussien et al (2010) | - Leaves, seeds and roots of *Coleus abyssinicus (Fresen.) A.J.Paton* , collected from its natural environment in Ethiopia, 12.5, 25 and 50 µg/µL - Leaves, seeds and fruits of *Pycnostachys eminii Gürke,* 12.5, 25 and 50 µg/µL | Y – essential oil extraction, compound isolation and antibacterial activity was done using method previously reported | Y - GC-MS |
| Delelegn et al (2018) | - Leaves of *Moringa oleifera Lam.,* obtained from Agricultural research centre in Ethiopia, 50 mg/mL | Y - plant material extraction and antibacterial activity testes were done using methods reported before | N |
| Habbal et al (2011) | - Leaves of *Lawsonia inermis L.*, collected from its natural habitat in Jordan, 50%, 25%, 12.5% | Y – extraction and antimicrobial activity tests were done using standard protocols | N |
| Nagarajan et al (2013) | - Leaves of *Lawsonia inermis L.*, collected from its natural environment in India, 1000, 2000, 3000 and 4000 µg/µL | Y - plant material extraction and antibacterial activity testes were done using methods reported before | N |
| Maleki et al (2018) | - Leaves of *Azadirachta indica A.Juss.*, collected from its natural habitat in Iran, 300, 200, 100, and 50 mg/mL | Y - extraction and antibacterial activity was performed using standard protocols | N |
| Mohammed et al (2018) | - Leaves of *Azadirachta indica A.Juss.*, collected from it natural environment in Sudan*,* 100 mg/ml, 50mg/ml, 25mg/ml, 12.5mg/ml, and6.25mg/ml | Y - extraction and antibacterial activity was performed using standard protocols | N |
| Reddy et al, 2013 | - Leaves, bark and fruits of *Azadirachta indica A.Juss.*, collected from its natural environment in India, 500, 1000 and 2000µg/ml | Y - extraction and antibacterial activity was performed using standard protocols | N |

**Table 2 - Botanical or multiherbal for antifungal studies**

| **Study** | **Species, sources, concentrations** | **Quality control reported (Y/N)** | **Chemical analysis reported (Y/N)** |
| --- | --- | --- | --- |
| Asres et al (2006) | - Stem bark *Combretum molle R.Br. ex G.Don,* collected from Gual Mereb in Tigray region, Ethiopia 100 to 2000 mg/m | Y- extraction using Soxhlet apparatus and extracts were tested using previously reported methods | N |
| Sileshi et al (2007) | - Leaves of *Rotheca myricoides (Hochst.) Steane & Mabb.*, collected from its natural habitat in Ethiopia, 25, 50 and 100 mg/ml - Leaves of *Ficus palmata Forssk.*, collected from its natural habitat in Ethiopia, 25, 50 and 100 mg/ml - Leaves of *Grewia ferruginea Hochst. ex A.Rich.* , collected from its natural habitat in Ethiopia, 25, 50 and 100 mg/ml - Aerial parts of *Periploca linearifolia Quart.-Dill. & A.Rich.,* collected from its natural habitat in Ethiopia, 25, 50 and 100 mg/ml | Y -plant materials extraction and antifungal activity was performed using standard methods | N |
| Vaijayanthimala et al (2000) | - Clove of *Allium sativum L.* - , purchased from Ayurvedic medical shop in India, 300 mg/mL - Clove of *Allium schoenoprasum* L, purchased from Ayurvedic medical shop in India, 300 mg/mL - Bulbs of *Allium cepa var. cepa* L, purchased from Ayurvedic medical shop in India, 300 mg/mL - Leaves of *Acalypha indica* L, purchased from Ayurvedic medical shop in India, 300 mg/mL - Leaves and seeds *Azadirachta indica A.Juss.*, purchased from Ayurvedic medical shop in India, 300 mg/mL - Leaves of *Camellia sinensis var. kucha Hung T.Chang & S.S.Wang*, purchased from Ayurvedic medical shop in India, 300 mg/mL - Leaves of *Cassia alata* L., purchased from Ayurvedic medical shop in India, 300 mg/mL - Leaves of *Cassia fstula* L., purchased from Ayurvedic medical shop in India, 300 mg/mL - Leaves of *Cassia occidentalis* L, purchased from Ayurvedic medical shop in India, 300 mg/mL - Seeds of *Coffea arabica* L, purchased from Ayurvedic medical shop in India, 300 mg/mL - Roots of *Curcuma longa*L*.*, purchased from Ayurvedic medical shop in India, 300 mg/mL - Leaves of  *Lawsonia inermis L.,* purchased from Ayurvedic medical shop in India, 300 mg/mL - Leaves of *Murraya koenigii (L.) Spreng.*, purchased from Ayurvedic medical shop in India, 300 mg/mL - Leaves of *Ocimum sanctum* L, purchased from Ayurvedic medical shop in India, 300 mg/mL - Leaves of *Piper betle* L, purchased from Ayurvedic medical shop in India, 300 mg/mL - Seeds of *Psoralea corylifolia* L, purchased from Ayurvedic medical shop in India, 300 mg/mL | Y -plant materials extraction and antifungal activity was performed using standard methods | N |
| Bora et al (2016) | - Seeds of *Cinnamomum parthenoxylon (Jack) Meisn.,* collected from its natural habitat in India, 0.2 mg/mL - Leaves of *Phyla nodiflora (L.) Greene,* collected from its natural habitat in India, 0.2 mg/mL - Fruit of *Cestrum nocturnum L.,* collected from its natural habitat in India, 0.2 mg/mL - Seeds of *Trachyspermum ammi (L.) Sprague,* collected from its natural habitat in India, 0.2 mg/mL - Fruit of *Sida planicaulis Cav.,* Collected from its natural habitat in India, 0.2 mg/mL | Y -plant materials extraction and antifungal activity was performed using standard methods | N |
| Park et al (2007) | - Essential of *Leptospermum petersonii F.M.Bailey,* G. R. Davis PTY. Ltd. Co., Australia, 0.05, 0.1, 0.15, and 0.2 mg/ml - Essential of *Syzygium aromaticum (L.) Merr. & L.M.Perry,* purchased from Dullberg Konzentra Co., Germany, 0.05, 0.1, 0.15, and 0.2 mg/ml | Y - antifungal activity was performed using standard methods | Y - GC/MS |
| Ameya et al (2016) | - Roots of Echinops kebericho Mesfincollected from its habitat in Ethiopia, 200 µg/ml, 1.56-100µg/ml | Y - plant material extraction and antifungal activity was tested using standard methods | N |
| Ameya et al (2015) | - Roots of *Taverniera abyssinica A.Rich.,* purchased from a local traditional medicine vendor shop in Ethiopia, 10, 20, 40 and 80 mg/mL | Y - plant material extraction and antifungal activity was tested using standard methods | N |
| Gemeda et al (2014) | - Aerial part of *Cymbopogon martini (Roxb.) W.Watson,* collected from a botanical garden in Ethiopia, - Aerial part of *Foeniculum vulgare Mill.*, collected from garden of a farmer in Ethiopia, - Seeds of *Trachyspermum ammi (L.) Sprague,* collected from a garden of a farmer in Ethiopia, | Y – essential oil extraction and antifungal activity testes were done using standard methods | Y – HPLC/TLC |
| Rana et al (2011) | - Leaves of *Syzygium aromaticum (L.) Merr. & L.M.Perry,* purchased from a local market in India, 0.2% | Y – essential oil extraction and antifungal activity testes were done using standard methods | Y – HPLC/TLC |
| Getie et al (2003) | - Leaves of *Dodonaea viscosa Jacq.,* collected from its natural habita in Ethiopia, 25, 50, 100 mg/ml - Leaves of *Rumex nervosus Vahl,* purchased from a local market in Ethiopia, 25, 50, 100 mg/ml - Roots of *Rumex abyssinicus Jacq.,* purchased from a local market in Ethiopia, 25, 50, 100 mg/ml | Y - plant materials extraction and antifungal activity tests were performed using standard methods | N |
| Fierascu et al (2018) | - Fruit of  *Juniperus communis L.,* collected from its natural habitat in Romania, 50 μL per well | Y - plant materials extraction and antifungal activity tests were performed using standard methods | UV-Vis spectrometry and Chromatography |
| Nyanchok (2007) | - Stem bark of *Bersama abyssinica Fresen.,* collected from its natural habitat in Kenya, 500µg/disc | Y – extraction, fractionation, compound isolation and anti-bacterial activities were done according to methods previously reported | NMR, HPLC |
| Jain et al (2017) | - Essential oil of *Thymus vulgaris L.,* purchased from Sigma Aldrich, Brazil | Y – antifungal activity was performed using standard methods | N |
| Messele et al (2004) | - Leaves and fruit of *Pentanema confertiflorum (A.Rich.) D.Gut.Larr., Santos-Vicente, Anderb., E.Rico & M.M.Mart.Ort.,* collected from its natural habitat in Ethiopia, 40, 20, 10, 5, 2.5 and 1.25 mg/ml - Leaves of *Clematis simensis Fresen.,* collected from its natural habitat in Ethiopia, 40, 20, 10, 5, 2.5 and 1.25 mg/ml - Leaves of *Zehneria scabra (L.f.) Sond.,* collected from its natural habitat in Ethiopia, 40, 20, 10, 5, 2.5 and 1.25 mg/ml - Leaves of *Pycnostachys abyssinica Fresen.,* collected from its natural habitat in Ethiopia, 40, 20, 10, 5, 2.5 and 1.25 mg/ml | Y- plant material extraction and antifungal activity tests were performed according to standard methods | Y – preliminary phytochemical screening was performed |
| Salazar et al (2015) | - Leaves of *Azadirachta indica A.Juss.,* collected from its natural environment in Colombia, 0.0019 to 7000 μg/mL | Y – Extraction, compound isolation and antifungal activity was performed using standard methods | Y- HPLC |
| Simhadri et al (2017) | - Leaves of *Azadirachta indica A.Juss.,* collected from its natural habitat in India, 1mg/ml | Y- extraction and antifungal activity testes were performed using standard methods | N |

**Table 3 – Isolated chemical compound**

| **Study** | **Compound, Concentration** | **Sources** | **Purity (%) (and grade, if applicable)** | **Quality control reported? (Y/N)** |
| --- | --- | --- | --- | --- |
| Oumer et al (2014) | - Pure compound (Aloin A/B), 200 μg/mL - Pure compound (Aloin-6'-O-acetae A/B), 200 μg/mL | Purified by Oumer et al (2014) | NMR | Y |
| Chaieb et al (2011) | - Pure compound (Thymoquinone), 0 to 512 μg/ml | purchased from Sigma-Aldrich, Switzerland | ≥ 98% | Y |
| Awino et al (2007) | - Pure compound (compound 1), 100mM | Purified by Awino et al (2007) | NMR | Y |
| Duraipandiyan et al (2012) | - Pure compound (Costunolide), 250 μg/ml - Pure compound (Eremanthin), 250 μg/ml | Purified by Duraipandiyan et al (2012) | GC-MS | Y |
| Megeressa et al (2015) | - Pure compound (Aloesin), - Pure compound (8-O-Methyl-7-hydroxyaloin A/B), - Pure compound (Aloin A/B (3)) - Pure compound (Aloin-6’-O-acetate A/B) | Purified by Megeressa et al (2015) | HPLC | Y |
|  |  |  |  |  |

**Table 4: Formulations based national monographs (‘patented’ formulations), botanical or chemical**

| **Study** | **Formulation** | **Source** | **Species, concentration** | **Quality control reported? (Y/N)** | **Chemical analysis reported? (Y/N)** |
| --- | --- | --- | --- | --- | --- |
| Kasparaviciene et al (2018) | Oleogel (concentration of 0.1% w/w) | Sigma-Aldrich Chemie GmbH and Carl Roth GmbH & Co. Kg | Thyme, 0.01 to 0.3% | Y – used inhouse method | Y - GC-FID analyses were |
